# Mannich bases derived from lawsone and their metal complexes: synthetic strategies and biological properties

**DOI:** 10.1039/d0ra05717g

**Published:** 2020-08-17

**Authors:** Abolfazl Olyaei, Mahdieh Sadeghpour, Mehdi Khalaj

**Affiliations:** Department of Chemistry, Payame Noor University (PNU) PO BOX 19395-4697 Tehran Iran olyaei_a@pnu.ac.ir; Department of Chemistry, Takestan Branch, Islamic Azad University Takestan Iran; Department of Chemistry, Buinzahra Branch, Islamic Azad University Buinzahra Iran

## Abstract

Lawsone (2-hydroxynaphthalene-1,4-dione) is a natural product which shows significant biological activity. Aminomethylnaphthoquinone Mannich bases derived from lawsone constitute an interesting class of naphthoquinones and/or their metal complexes have demonstrated a series of important biological properties. So, this review aimed to document the publications concerning the synthesis of aminomethylnaphthoquinone Mannich bases from lowsone, aldehydes and amines and their metal complexes using different conditions, and investigation of their applications.

## Abstract 2

Lawsone (2-hydroxynaphthalene-1,4-dione) is a natural product which shows significant biological activity.

## Graphical abstract

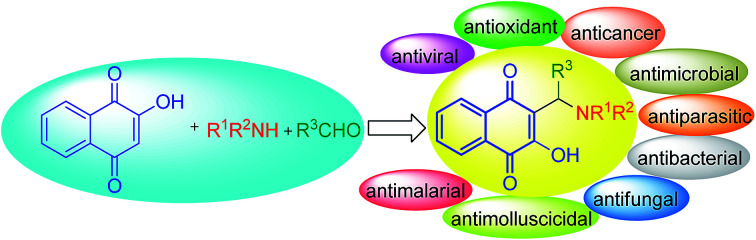

## 1. Introduction

Molecules with the quinoid structure bearing a hydroxy group on the quinone ring constitute one of the most interesting classes of synthetic compounds in organic chemistry and they are widely distributed in nature. Specifically, naphthoquinone structures receive great attention because of their pharmacological activities such as antibacterial, antiviral and antifungal activities.^1–3^ 2-Hydroxy-1,4-naphthoquinone, or lawsone, or hennotannic acid, has been known for the past 4000 years, is one of the simplest naturally occurring naphthoquinones. It is a red-orange dye, classified as Natural Orange 6 (C.I.75480), present in the leaves of the henna plant (*Lawsonia* spp., family Lythraceae) as well as in the flower of water hyacinth (*Eichhornia crassipes*) and in jewelweed *Impatiens balsamina.*^4,5^ It is traditionally used for coloring hair and dying nails, skin, wool and cotton.^6–10^ Besides it has several other uses including antiaging additive to vulcanized natural rubber ,^11^ skin protection from ultraviolet radiation,^12,13^ oxidation of chlorinated compounds,^14,15^ corrosion inhibition for steel,^16,17^ and sensitive colorimetric and electrochemical sensor for anions.^18^ Extracts of henna are widely used in folk medicine to treat burn wounds infected by microorganisms^19^ due to its antibacterial properties^20^ as well as headaches, lumbago, bronchitis, ophthalmia, syphilis, sores and amenorrhoea.^21^ The classical Mannich reaction is one of the most important carbon–carbon bond-forming reactions in organic synthesis because of its atom-economy advantages and application in biologically active molecule syntheses. However, a three-component condensation between structurally diverse substrates containing at least one acidic hydrogen atom, an aldehyde component and an amine reagent leads to Mannich bases. This multicomponent reaction usually occurs under acid catalysis, although catalysis is not mandatory.

3-(Aminomethyl)-2-hydroxy-1,4-naphthoquinones known as aminonaphthoquinone Mannich bases, constitute an interesting class of naphthoquinones and/or their metal complexes have demonstrated a series of important biological properties such as anticancer,^22^ antimalarial,^23^ antimolluscicidal,^24^ antibacterial,^25^ cholinesterase inhibitors,^26^ leishmanicidal,^27^ antiviral,^28^ antifungal,^29^ anti parasitic^30^ activities and they have also been employed as fluorescent compounds and dyes.^31^ Because of their prevalent applications, several methods have been reported for the synthesis of these important compounds and their metal complexes. This review will summarize the reported methods for the synthesis of aminonaphthoquinone Mannich base derivatives by multicomponent reactions of lawsone with a non-enolizable aldehyde and a primary or secondary amine *via* the Mannich reaction and their metal complexes using different conditions, and applications of these compounds.

## 2. Synthesis of aminomethylnaphthoquinone Mannich bases

### 2.1. Uncatalyzed reactions

**Scheme 1:**
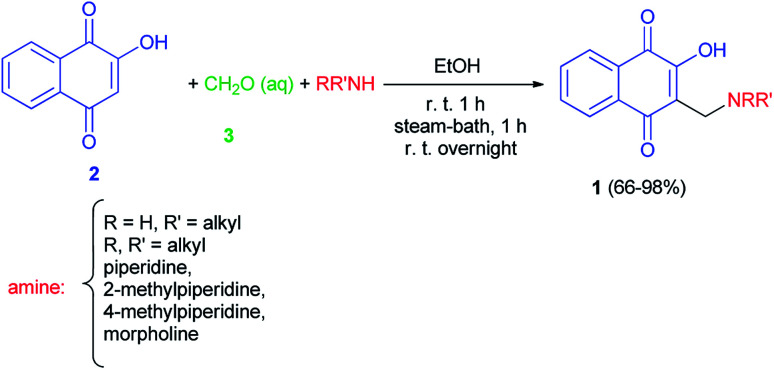
Synthesis of 2-hydroxy-3-aminomethyl-1,4-naphthoquinones 1.

The first synthesis of 2-hydroxy-3-aminomethyl-1,4-naphthoquinones 1 by the Mannich reaction has been reported by Leffler and Hathaway in 1948. The reaction mixture of lawsone (2), amine and 37% formalin (3) in absolute ethanol was allowed to stir at room temperature for one hour was warmed on the steam-bath for an additional hour, and was then left at room temperature overnight gave the corresponding aminonaphthoquinone 1 in 66–98% yields. In this study diethylamine failed to react, on the other hand, primary amines, such as butylamine, gave especially good yields (Scheme 1).^32^

**Scheme 2:**
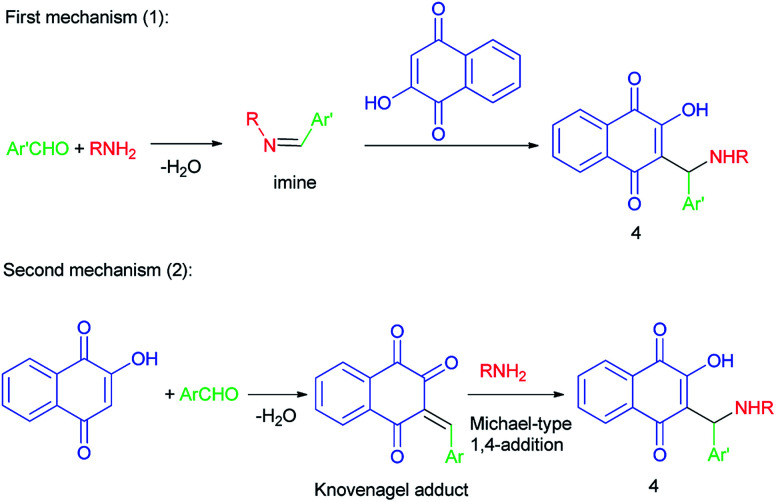
Proposed mechanisms for the synthesis of aminomethylnaphthoquinones 4.

However, two mechanisms may be operating in the formation of the aminomethylnaphthoquinone derivatives (4), depending on the reaction conditions: the first mechanism is preformation of the imine *via* the reaction of amine and aldehyde followed by nucleophilic attack of lawsone (2) to the activated imine or iminium species, and/or the second mechanism is Knovenagel-type condensation of lawsone with aldehyde followed by Michael-type 1,4-addition of amine to the alpha, beta-conjugated carbonyl species formed. Apparently, mechanism (1) operates mostly in catalyzed transformations while mechanism (2) will probably be the one operating for uncatalyzed transformations (Scheme 2).

**Scheme 3:**
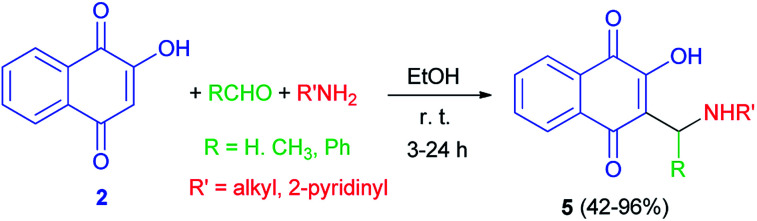
Synthesis of aminonaphthoquinines 5.

In 1949, Dalgliesh demonstrated that Mannich bases 5 were prepared by the reaction of lawsone, aldehydes and primary aliphatic amines or 2-aminopyridine in EtOH at room temperature for 3–24 h. The mechanism of the Mannich reaction is still obscure, it being uncertain whether the primary reaction is that of formaldehyde with the amine or with the active hydrogen component (Scheme 3).^33^ It has been suggested that the formation of aminonaphthoquinine 5 was occurred through the second mechanism as shown in Scheme 2.

**Fig. 1:**
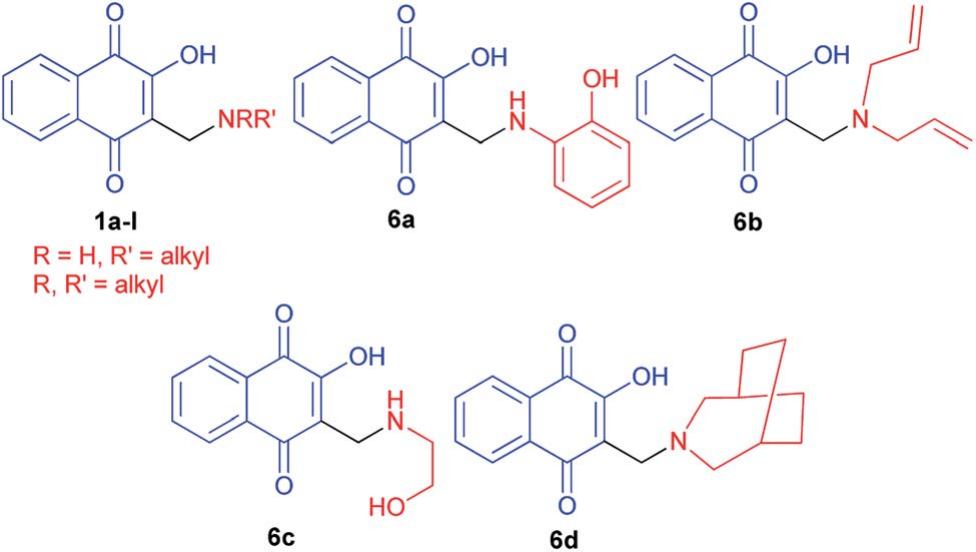
Structures of the synthesized hydroxynaphthoquinones 1 and 6a–d.

Hydroxynaphthoquinones 1 and 6a–d^24^ were synthesized as previously described methods (Fig. 1).^32,34^ They have been submitted to molluscicidal bioassays against the snail *Biomphalaria glabrata*, intermediate host of *Schistosoma mansoni*. Several of the quinones 1 and 6a–d assayed showed significant molluscicidal activities, and correlation of their activities and electrochemical parameters showed that the first wave reduction potential is an important parameter. The easily reduced quinones (>*E*_p1c_) were more active against adult snails and against their egg masses, whilst the 3-methylamino-2-hydroxy derivatives presented higher negative reduction potentials and were not active as molluscicides.

**Scheme 4:**
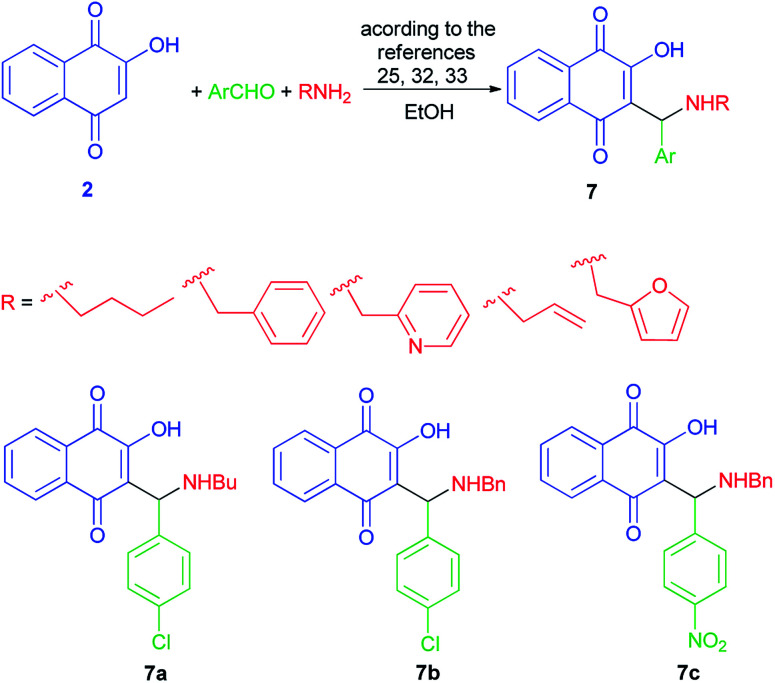
Synthesis of aminomethylnaphthoquinone derivatives 7.

Aminomethylnaphthoquinones 7 were synthesized from the Mannich reaction of lawsone (2) with the desired substituted benzaldehydes and primary amines in ethanol as described previously,^32,33^ with some modifications (Scheme 4).^25^ Although AMNQs 7a–c exhibited similar antiviral activities against BoHV-5, AMNQ 7c was at least 50-fold less cytotoxic than the other two compounds.^3^

**Scheme 5:**
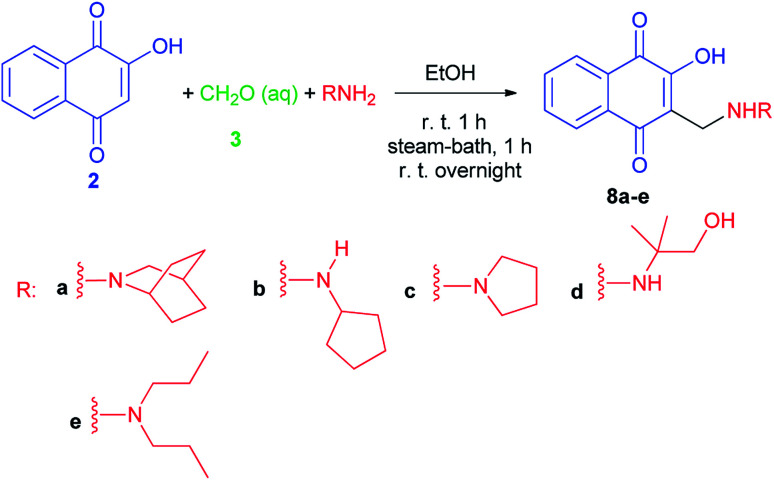
Naphthoquinones 8 obtained by Mannich reaction from lawsone.

2-Hydroxy-3-substituted-aminomethyl naphthoquinones 8a–e,^35^ Mannich adducts, were obtained in high quantities by the classic reaction of lawsone (2), using amine and formaldehyde in alcoholic solution (Scheme 5), as previously reported by the Pinto group,^24,36,37^ based on the procedure described by Leffler and Hathaway.^32^ Compounds 8 exhibit molluscicidal activity^24,36,37^ against the snail *Biomphalaria glabrata*, the causative agent of schistosomiasis. Compound 8e, the most active member of this group, was two times less effective than benznidazole (IC_50_/24 h = 103.6 ± 0.6 μM), the standard anti-*T. cruzi* drug, and 2.6 times more active than crystal violet (IC_50_/24 h = 536.0 ± 3.0 μM), which is recommended by WHO to hemotherapeutic centers in endemic areas for eliminating the parasite in blood used for transfusions.^35^

**Fig. 2:**
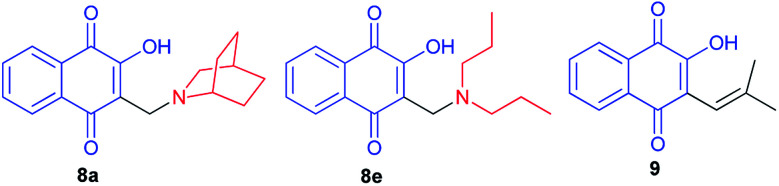
Structure of the naphthoquinones derived from lawsone (8a,e and 9).

Krettli *et al.* noted that the naphthoquinones derived from lawsone (compounds 8a and 8e) showed the highest activity against *P. falciparum* chloroquine-resistant blood-stage parasites (clone W2), indicated by their low inhibitory concentration for 50% (IC_50_) of parasite growth. The therapeutic potential of the compounds was evaluated according to the selectivity index, which is a ratio of the cytotoxicity minimum lethal dose which eliminates 50% of cells and the *in vitro* IC_50_. Naphthoquinones 8a and 8e, with activities similar to the reference antimalarial chloroquine, were also active against malaria in mice and suppressed parasitaemia by more than 60% in contrast to Nor-lapachol (9) which was inactive (Fig. 2).^38^

**Scheme 6:**
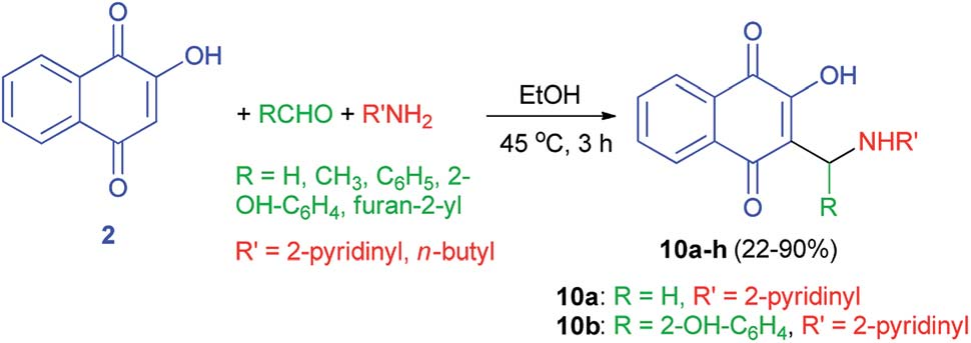
Synthesis of 2-hydroxy-1,4-naphthoquinones 10.

A series of substituted derivatives of 2-hydroxy-1,4-naphthoquinones 10 were prepared by a Mannich reaction from lawsone (2), aryl/alkyl aldehydes and 2-aminopyridine/*n*-butylamine in EtOH at 45 °C for 3 h in 22–90% yields (Scheme 6). Minimum inhibitory concentration (MIC) of the synthesized compounds was measured against *Mycobacterium tuberculosis* (MTB) H37Ra strain. Among them, two compounds 10a,b showed antibacterial activity within the MIC range of 20–50 μg mL^−1^ while the other compounds did not show antibacterial activity. It was found that compound 10a showed the high activity anti-tuberculosis among other compounds, although cytotoxicity issues have been observed when evaluated on Verocells for further concerns.^39^

**Scheme 7:**
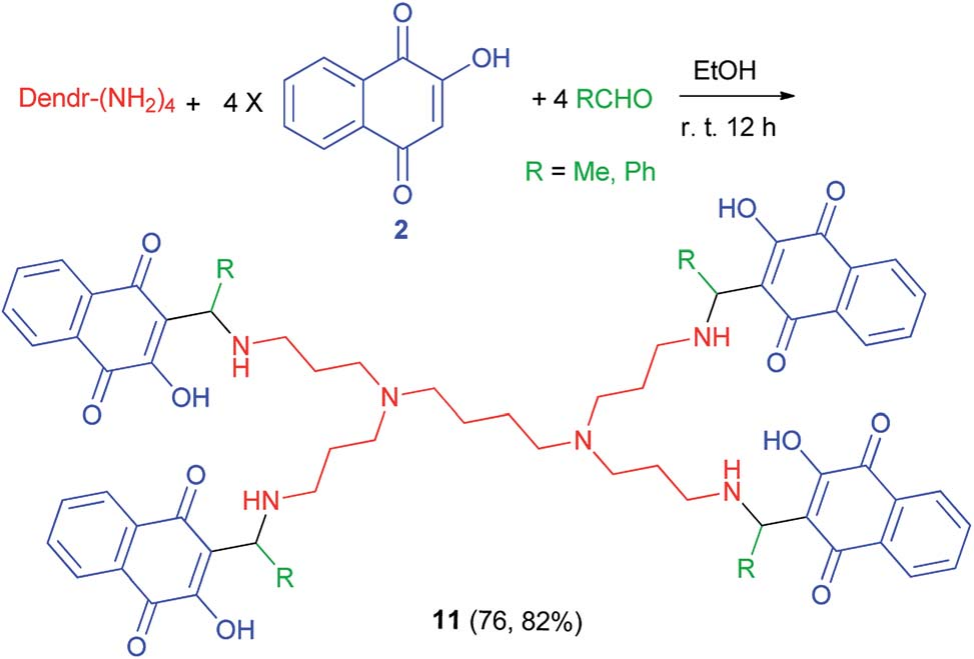
Synthesis of PPI-G1 dendrimers containing lowsone 11.

The synthesis of two new PPI-G1 dendrimers containing lowsone 11 as pharmacophoer is reported by Ndiaye and co-workers. The compounds were synthesized in course of Mannich reaction involving lawsone (2), the commercial PPI-G1(polypropyleneimine-G1) dendrimer with functional amino groups on the exterior and acetaldehyde or benzaldehyde in 4 : 1 : 4 molar ratio in absolute EtOH at room temperature for overnight in the dark (Scheme 7). The reaction mechanism could be explained briefly in a few words, *i.e.* condensation of the amino with the acidified aldehyde to give the imine followed by protonation and addition of lawsonate anion (mechanism (1)).^40^

**Scheme 8:**
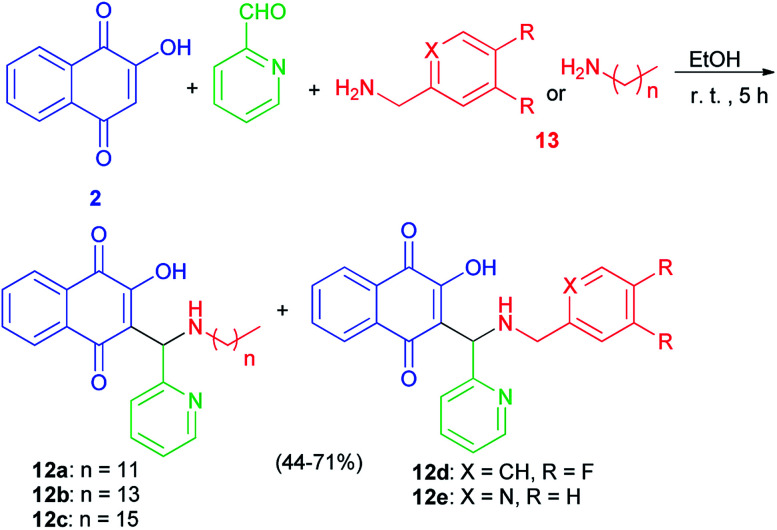
Synthesis of Mannich bases 12a–e.

**Scheme 9:**
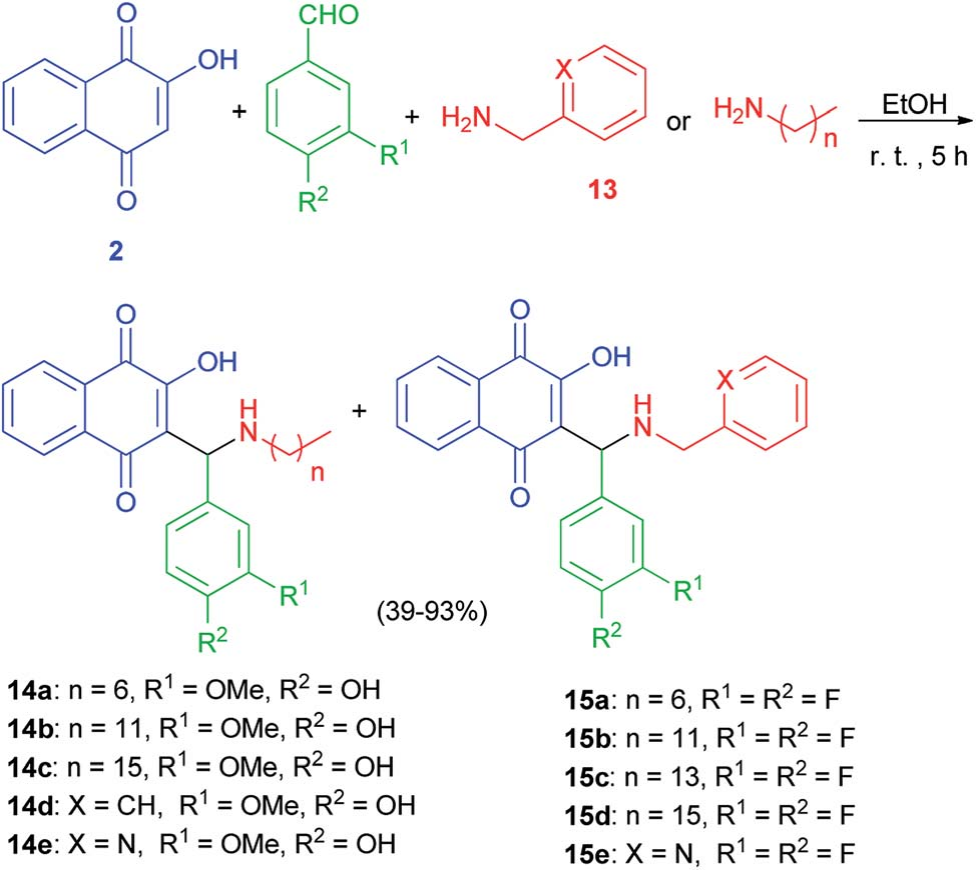
Synthesis of Mannich bases 14 and 15.

The Mannich bases 12a–e were prepared in 44–71% yields *via* Mannich reaction of lawsone, 2-pyridylcarboxaldehyde and the corresponding amines 13 in detail, dodecyl amine, tetradecyl amine, hexadecyl amine, 3,4-difluorobenzylamine and 2-pyridylmethylamine in EtOH at room temperature for 5 h (Scheme 8). In addition, closely related vanillyl and 3,4-difluorophenyl derivatives 14a–e and 15a–e were prepared. Analogously to the synthesis of compounds 12a–e, the Mannich reaction of lawsone with vanillins and the corresponding primary amines in EtOH gave the Mannich bases 14a–e as orange-red solids. In addition, reaction of lawsone with 3,4-difluorobenzaldehyde and various primary amines led to compounds 15a–e (orange-red solids). These target compounds were obtained in EtOH at room temperature in moderate to high yields (39–93%) (Scheme 9). Substituted lawsone Mannich bases 12a–e, 14a–e and 15a–e tested for their biological activities. The new fatty alkyl substituted compounds 12a–c exhibited strong and selective growth inhibitory activities in the low one-digit micromolar and sub-micromolar range against a panel of human cancer cell lines associated with ROS formation. In addition, compounds 12a–c revealed sub-micromolar anti-trypanosomal activities against parasitic *Trypanosoma brucei* cells *via* deformation of the microtubule cytoskeleton. The *N*-hexadecyl compound 12c was also highly active against locally isolated *Entamoeba histolytica* parasite samples exceeding the activity of metronidazole.^30^

**Scheme 10:**
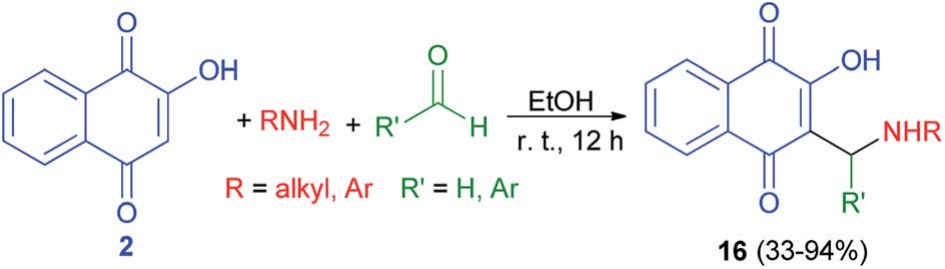
Synthesis of 3-(aminomethyl)naphthoquinones 16.

The synthesis of 3-(aminomethyl)-2-hydroxy-1,4-naphthoquinones 16 was carried out following a procedure described previously.^25^ The products were isolated as analytically pure orange powders with yields ranging from 33–94% (Scheme 10). In general, the compounds 16 have shown high to moderate activity against the HL-60 (promyelocytic leukaemia) cell line with IC_50_ = 1.1–19.2 μM. The results suggest that the nature of the aryl moiety introduced in the structure of products by the aldehyde component is crucial to the cytotoxicity, and although the group originated from the primary amine has a lesser influence, aromatic ones were found to suppress the activity.^41^

**Scheme 11:**
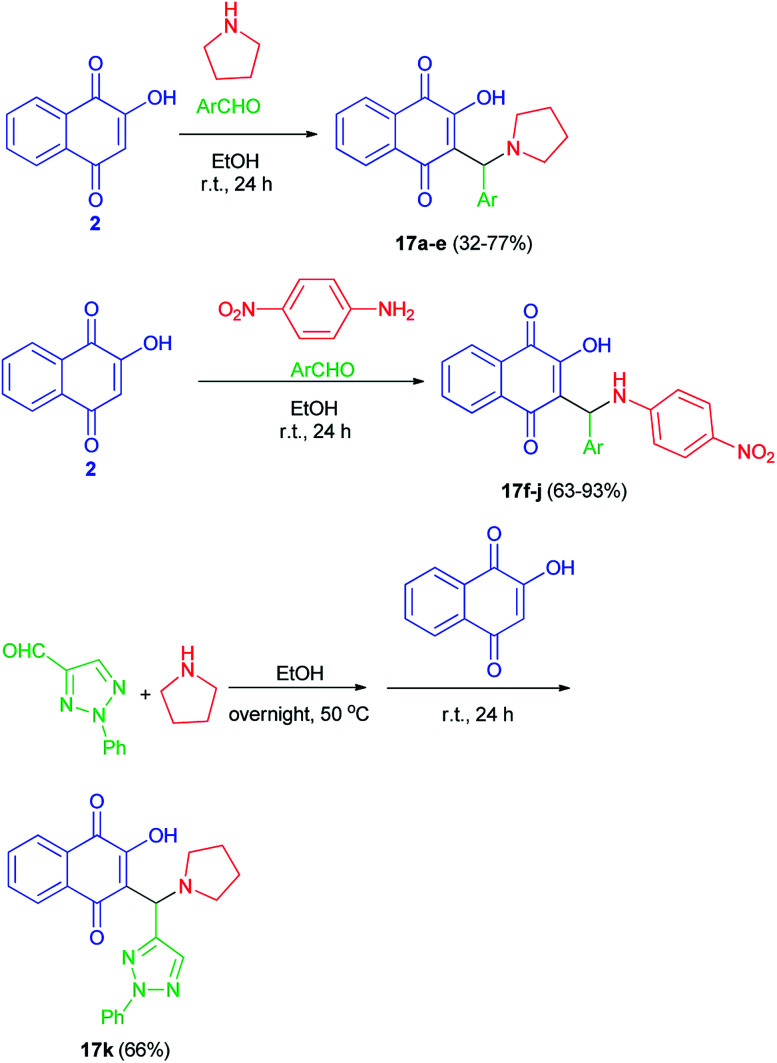
Synthesis of aminonaphthoquinone Mannich bases 17.

Greco *et al.* succeeded in preparation of aminonaphthoquinone derivatives 17 from lawsone (2), aldehydes and amines in EtOH at room temperature in the dark for 24 h. Also, for the synthesis of the lawsone derivative 17k, a mixture of 2-phenyl-2*H*-1,2,3-triazole-4-carbaldehyde and pyrrolidine in ethanol was stirred overnight at 50 °C. After this time, lawsone was added and the mixture was allowed to react for 24 h in the dark (Scheme 11). Among the synthetic lawsone derivatives, 2-hydroxy-3-((2-hydroxyphenyl)(pyrrolidin-1-yl)methyl)naphthalene-1,4-dione, 2-hydroxy-3-(((4-nitrophenyl)amino)(phenyl)methyl)naphthalene-1,4-dione and 2-hydroxy-3-((2-hydroxyphenyl)((4-nitrophenyl)amino)methyl)naphthalene-1,4-dione showed high activity against *Candida albicans* ATCC 10231, with minimal inhibitory concentrations (MICs) and minimal fungicidal concentrations (MFCs) ranging from 20 to 330 and from 80 to 330 μg mL^−1^, respectively. Moreover, they also showed a mechanism of action on exogenous ergosterol.^29^

**Scheme 12:**
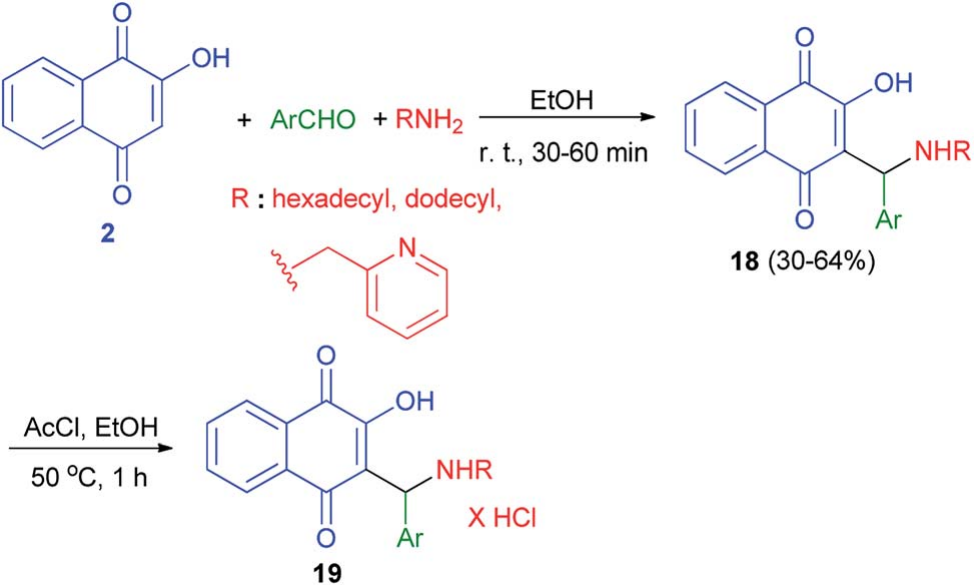
Synthesis of lawsone Mannich bases 18 and their hydrochloride salts 19.

Biersack *et al.* have been prepared a series of new lawsone Mannich bases 18 in 30–64% yields from lawsone (2), salicylaldehydes or nitrofurfural and dodecylamine or hexadecylamine or 2-aminomethylpyridine in EtOH at room temperature for 30–60 min. Compounds 18 were converted to the hydrochloride salts 19 by reaction with acetyl chloride in ethyl alcohol at 50 °C for 1 h (Scheme 12). These compounds tested for activities against *Leishmania major*, *Toxoplasma gondii*, and *Trypanosoma brucei* parasites. The hydrochloride salts 19 of the Mannich bases 18, derived from unsubstituted salicylaldehyde and long chained alkyl amines, were selectively and strongly active against *T. gondii* cells and appear to be new promising drug candidates against this parasite. Also, Some of them proved active against *L. major* promastigotes and up to four times more efficacious against *L. major* amastigotes.^42^

**Scheme 13:**
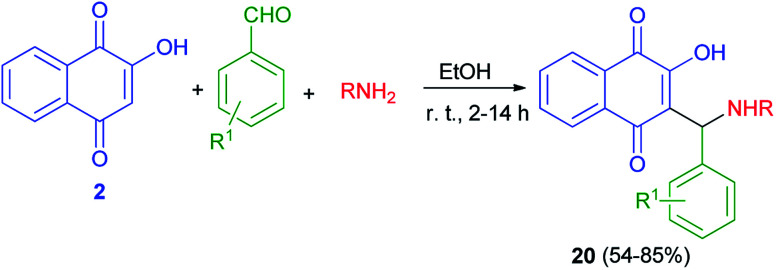
Synthesis of aminonaphthoquinone derivatives 20.

Thakore *et al.* described synthesis of Mannich bases of lawsone 20 by the reaction of lawsone, aromatic aldehydes and aliphatic amines in EtOH at room temperature for 2–14 h in 54–85% yields. It primarily forms the iminium ion by condensation of amine and aldehyde. Subsequently, the nucleophile lawsonate attacks iminium ion (analogous to the enolate) to give a Mannich base 20. Overall, the compounds with aliphatic substituent at R position and hydroxyl substituted phenyl ring at R^1^ position are associated with the highest activities. The effective IC_50_ concentration for compounds are 1.68–4.64 μM. These compounds showed higher anticancer potential against HepG2 cells (Scheme 13).^43^

**Scheme 14:**
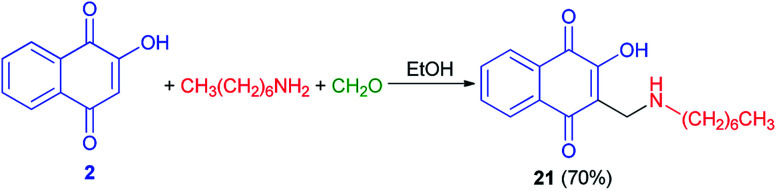
Synthesis of 3-(*N*-(1-heptyl)aminomethyl)-2-hydroxynaphtoquinone 21.

Moreover, compound 21 was obtained in 70% yield using a direct condensation of *n*-heptylamine on 2-hydroxynaphthoquinone (2) in the presence of formaldehyde in EtOH (Scheme 14). This novel atovaquone derivative 21 was tested for their *in vitro* activity against two apicomplexan parasites of medical importance, *Toxoplasma gondii* and *Plasmodium falciparum*, including resistant strains to atovaquone (*T. gondii*) and chloroquine (*P. falciparum*).^44^

### 2.2. Acid catalyzed reactions

**Scheme 15:**
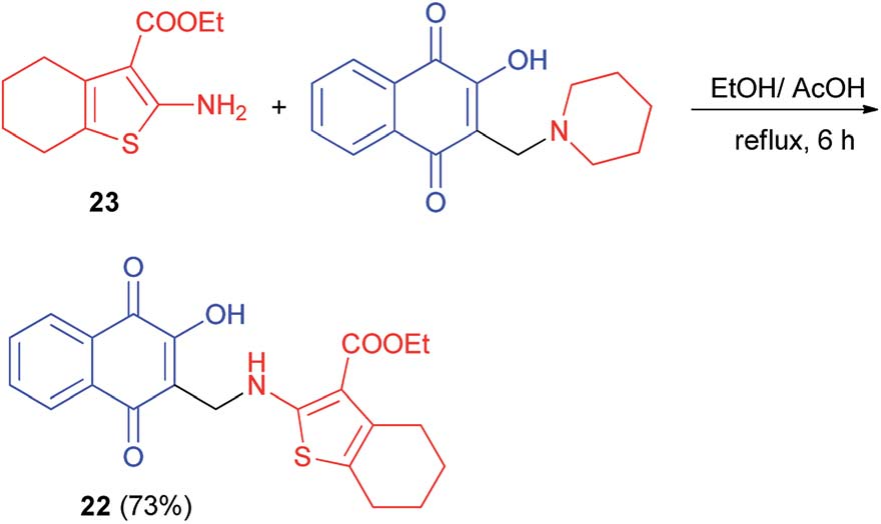
Synthesis of aminonaphthoquinone 22.

Synthesis of aminonaphthoquinone 22 in 73% yield has been carried out by the reaction of ethyl 2-amino-4,5,6,7- tetrahydrobenzo[*b*]thiophene-3-carboxylate 23 with 2-hydroxy-3-((piperidin-1-yl)methyl)naphthalene-1,4-dione in a mixture ethanol/acetic acid under reflux for 6 h (Scheme 15). It was screened for antioxidant activity. Calculation of the inhibition ratio (%) showed clearly that compound 22 exhibited moderate activity (63.17%) compared with ascorbic acid (89.87%) as a standard antioxidant. Also, it was selected for bleomycin-dependent DNA-damage testing. If the sample to be tested is able to reduce the bleomycin–Fe^3+^ to bleomycin–Fe^2+^, DNA degradation in this system will be stimulated, resulting in a positive test for pro-oxidant activity. The results showed that compound 22 has an ability to protect DNA from the induced damage by bleomycin. Thus, it would appear generally that introducing of naphthoquinone moiety enhances the antioxidant properties of 2-aminothiophene compounds.^45^

**Scheme 16:**
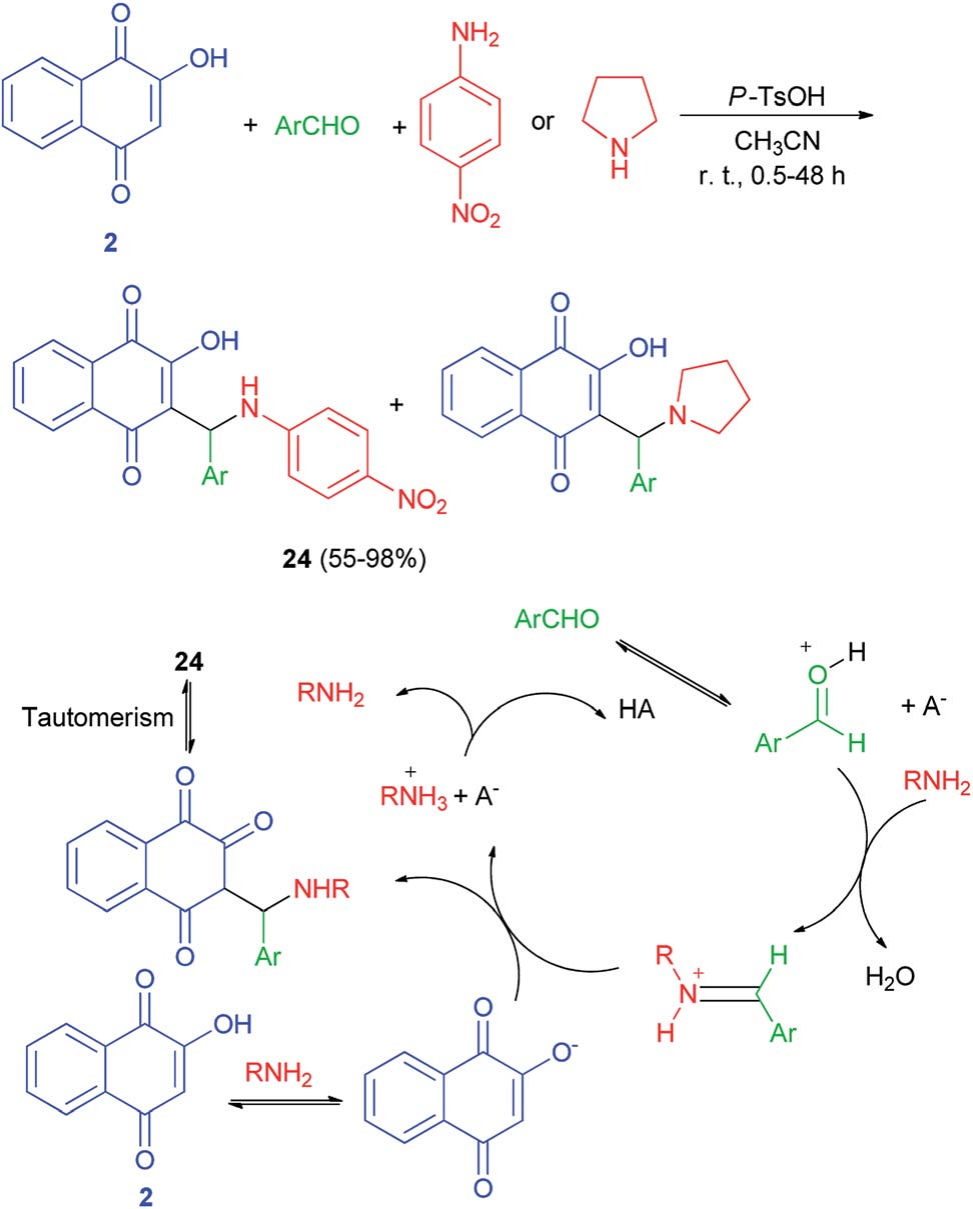
*p*-TsOH catalyzed synthesis of aminonaphthoquinones 24.

**Scheme 17:**
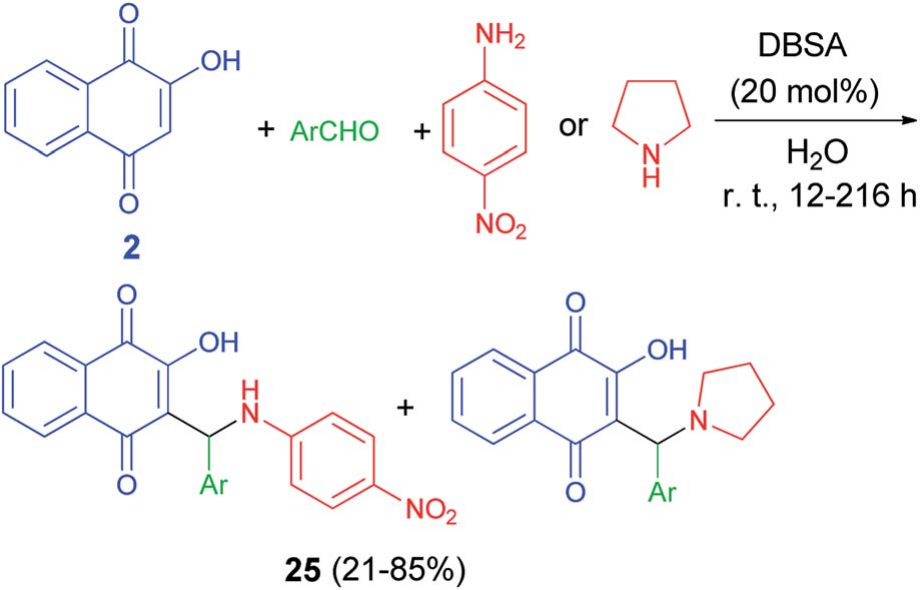
DBSA catalyzed synthesis of aminonaphthoquinones 25.

Greco *et al.* demonstrated that one-pot protocol was developed for the synthesis of a series of new aminonaphthoquinones 24 in 55–98% yields from 2-hydroxy-1,4-naphthoquinone (2), aldehydes and 4-nitroaniline or pyrolidine by three-component Mannich reaction using catalytic amount of *p*-TsOH in CH_3_CN at room temperature for 0.5–48 h. A reasonable mechanism possibility is shown in Scheme 16. First, the catalyst protonates the carbonyl oxygen of the aldehyde favoring the formation of the iminium ion which reacts with the nucleophilic species, that may be the ion formed from deprotonation of lawsone by the amine. Finally, the product is obtained after tautomerism and the catalyst is regenerated.^46^ Environmentally friendly one-pot protocol was developed for the first synthesis of aminonaphthoquinones 25 in 21–85% yields derived from lawsone (2) *via* a multi-component Mannich reaction in aqueous media using a catalytic amount of dodecyl benzenesulfonic acid (DBSA) (20 mol%), exploring a Brønsted acid-surfactant catalyst (BASC) concept, at room temperature for 12–216 h. Most likely, all of the reaction steps, from the iminium salt formation to the final Mannich adduct, occur inside of the surfactant micelles due to the affinity of the medium for reactants/intermediates (Scheme 17). In this context, DBSA enhances the chemical yield and decreases the reaction time by building a colloidal dispersion system and protonating electrophilic species, the aldehyde and imine, increasing its reactivity towards the nucleophile at the same time.^47^

**Scheme 18:**
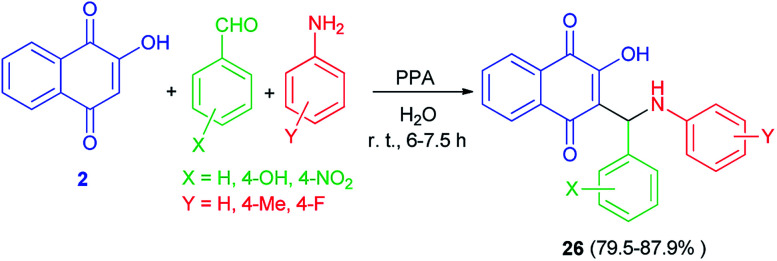
PPA catalyzed synthesis of hydroxylnaphthalene-1,4-dione derivatives 26.

An efficient one-pot synthesis of lawsone derivatives 26 in 79.5–87.9% yields is accomplished by a three-component reaction of 2-hydroxynaphthalene-1,4-dione (2), aromatic aldehydes and amines carrying electron-donating or electron-withdrawing substituents in water under ambient temperature catalyzed by phenylphosphinic acid for 6–7.5 h (Scheme 18). It should be noted that aliphatic aldehyde reacted poorly under the same conditions. The proposed mechanism for the formation of the compounds 26 is shown in Scheme 2 (mechanism (1)).^48^

**Scheme 19:**
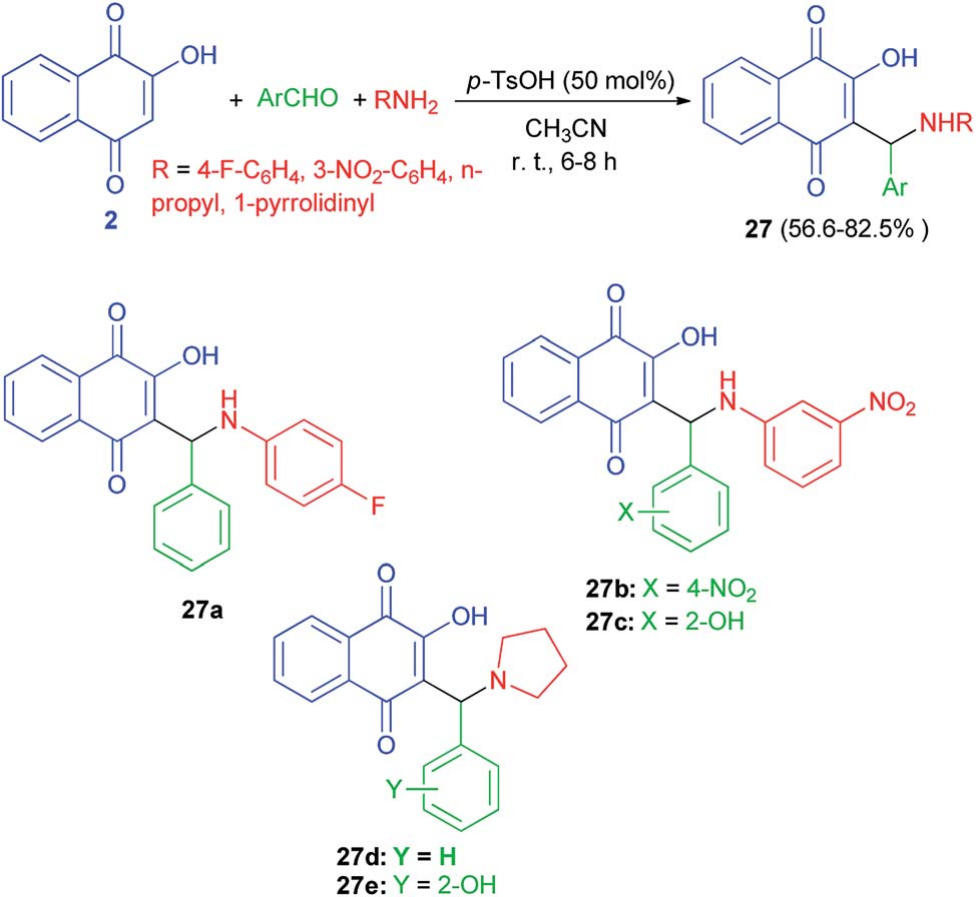
*p*-TsOH catalyzed Mannich bases of lawsone 27.

A series of Mannich bases of lawsone as 3-alkyl/aryl/heteroaryl substituted aminonaphthoquinones 27 were synthesized in 56.6–82.5% yields from lawsone (2), amines and aromatic aldehydes in CH_3_CN at room temperature in presence of *p*-TsOH (50 mol%) for 6–8 h (Scheme 19). Among synthesized compounds, five compounds (27a–e) showed good antimalarial activity against both chloroquine (CQ)-sensitive (3D-7) and -resistant (RKL-2) strains of *P. falciparum*, which, however, was considerably less than that of the standard reference drug, CQ. The antimalarial activity of these five compounds was found better against sensitive (IC_50_ = 0.411–0.502 μg mL^−1^) strain than the resistant (IC_50_ = 1.391–2.394 μg mL^−1^) one.^23^

**Scheme 20:**
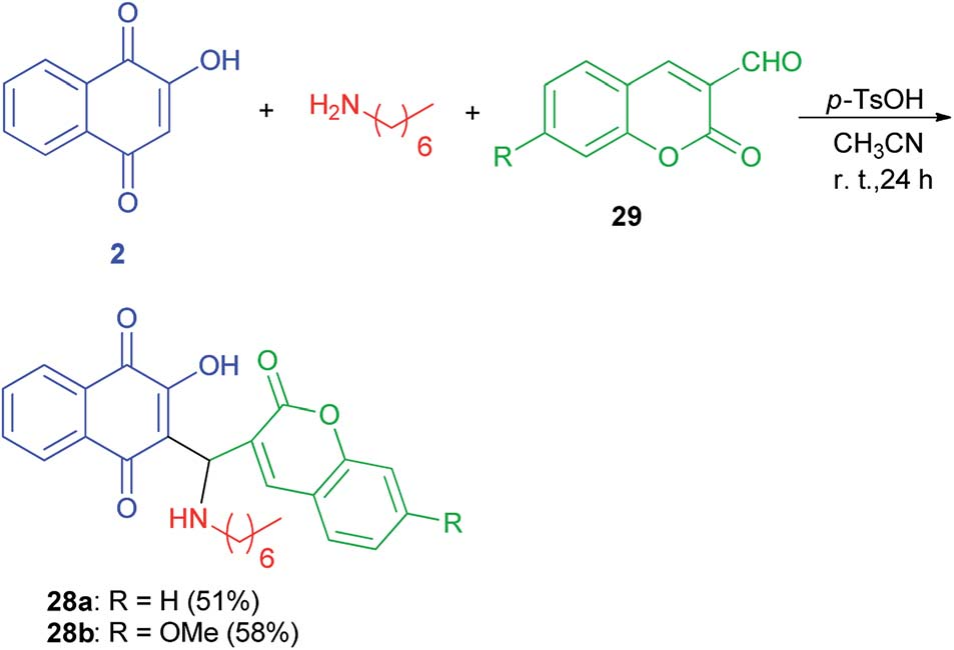
Synthesis of Mannich bases 28a–b.

Mannich bases 28a–b derived from lawsone, heptylamine and 3-formylcoumarins (29) have been synthesized in the presence of *p*-TsOH (20 mol%) in CH_3_CN at room temperature for 24 h in the dark in 51 and 58% yields, respectively (Scheme 20). The cyclic voltammetry data of these compounds showed a charge transfer (CT) process from the coumaryl to the naphthoquinonoid group, in spite of the absence of conjugation between these two fragments, with the nitrogen atom playing an important role.^49^

### 2.3. Metal catalyzed reactions

**Scheme 21:**
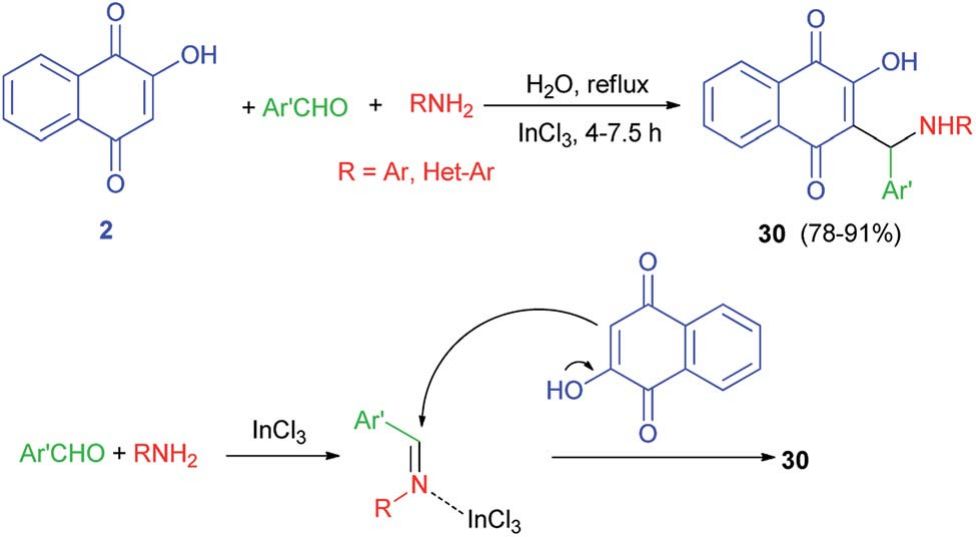
InCl_3_ catalyzed synthesis of aminonaphthoquinones 30.

An efficient one-pot synthesis of fluorescent hydroxyl naphthalene-1,4-dione derivatives 30 in 78–91% yields by atom-economical and three-component reaction of 2-hydroxynaphthalene-1,4-dione (2), aromatic aldehydes and heterocyclic or carbocyclic amines in the presence of a catalytic amount of InCl_3_ in refluxing water for 4–7.5 h is reported. The products were fluorescent in methanol solution emitting at green light (546–560 nm). Possible mechanism for the formation of products 30 as shown in Scheme 21.^31^

**Scheme 22:**
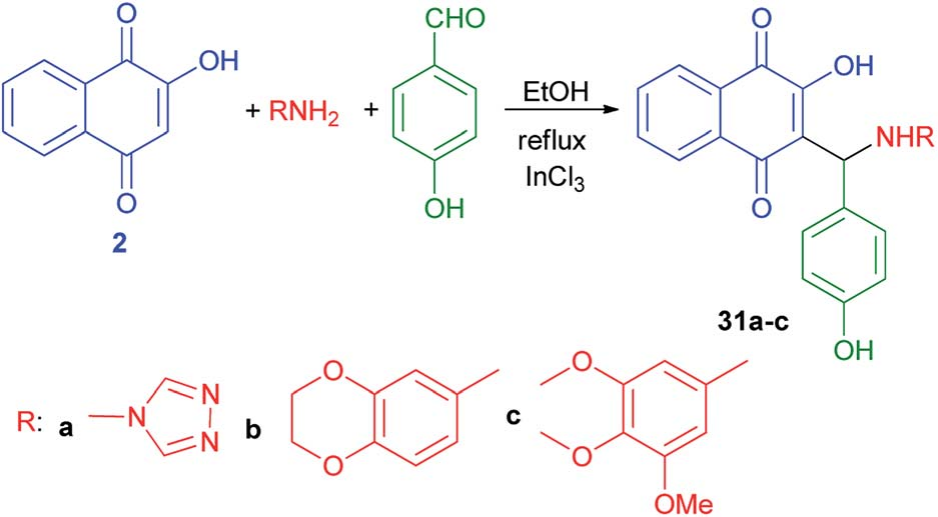
InCl_3_ Catalyzed synthesis of 2-arylaminonaphthoquinones 31a–c.

Ghodsi *et al.*^50^ have published synthesis of 2-arylaminonaphthoquinone derivatives 31a–c from lawson (2), 4-hydroxybenzaldehyde and aryl amines in the presence of InCl_3_ in refluxing EtOH according to the previously reported procedure (Scheme 22).^31^

**Scheme 23:**
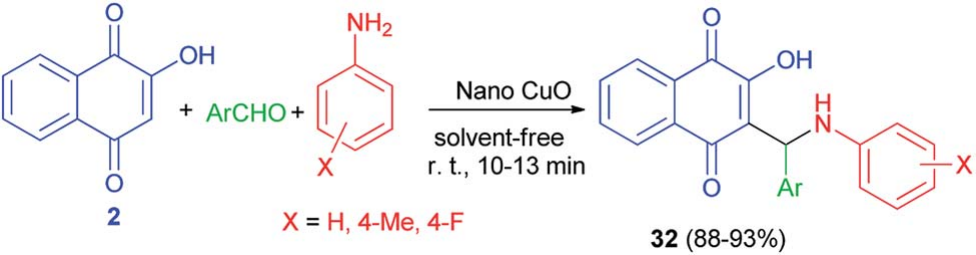
Nano CuO catalyzed synthesis of aminonaphthoquinone derivatives 32.

Aminonaphthoquinone derivatives 32 have been prepared in 88–93% yields by one-pot three-component condensation reaction of aromatic aldehydes, aromatic amines, and 2-hydroxynaphthalene-1,4-dione (2) in the presence of nano-copper(ii) oxide (8 mol%) as catalyst under mild, ambient and solvent-free conditions for 10–13 min. The proposed catalytic mechanism for the preparation of 32 using nano-copper(ii) oxide as the catalyst is described in Scheme 23.^51^ The proposed mechanism is similar to the first mechanism as shown in Scheme 2.

**Scheme 24:**
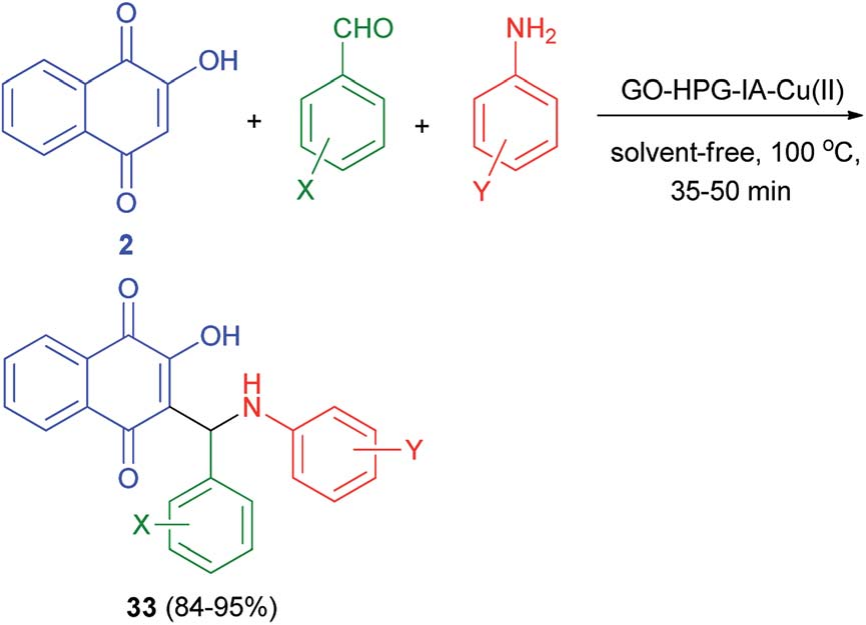
Synthesis of aminonaphthoquinone derivatives 33 using Cu(ii) immobilized on graphene oxide.

Cu(ii) immobilized on hyperbranched polyglycerol (HPG) functionalized graphene oxide catalyzed synthesis of aminonaphthoquinones 33 in 84–95% yields *via* one-pot three-component condensation reaction of lawsone (2), benzaldehyde and aniline derivatives at 100 °C for 35–50 min under solvent-free conditions (Scheme 24). According to the plausible mechanism, firstly, the aldehyde is activated by the GO–HPG–IA–Cu(ii) catalyst which upon nucleophilic attack of amine to form imine. Subsequently, lawsone attacks to imine in the presence of the GO–HPG–IA–Cu(ii) catalyst and undergoes tautomeric proton shift to generate the aminonaphthoquinone derivative 33 and releases the catalyst for the next run.^52^

**Scheme 25:**
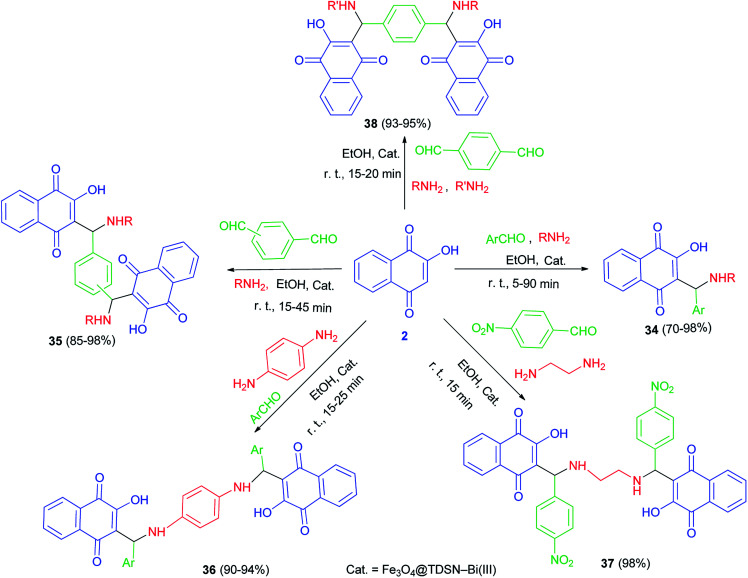
Synthesis of mono-and bis-aminonaphthoquinone derivatives 34–38.

**Scheme 26:**
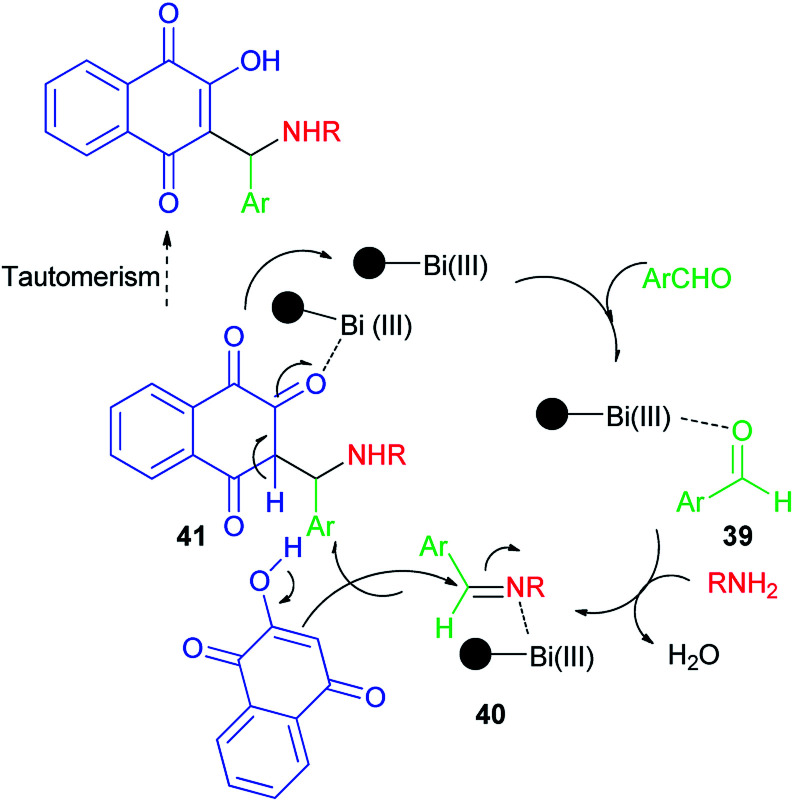
Plausible mechanism for the Fe_3_O_4_@TDSN–Bi(iii) catalyzed synthesis of aminonaphthoquinones 34–38.

Synthesis of mono-aminonaphthoquinone derivatives 34 catalyzed by Bi(iii) immobilized triazine dendrimer-stabilized magnetic nanoparticle Fe_3_O_4_@TDSN–Bi(iii) (3 mol%) *via* a one-pot three-component reaction of lawsone, aldehydes containing electron with-drawing or electron-donating groups and amines (anilines, 4-methylaminopyridines, *i*-butylamine, pyrolidine and piperidine) in EtOH as a green solvent at room temperature for 5–90 min in 70–98% yields. This catalytic system also showed excellent activity in the synthesis of symmetric and unsymmetric bis-aminonaphthoquinones 35–38 from dialdehyde (terephthaladehyde and isophthalaldehyde) and/or diamines (benzene-1,4-diamine and 1,2-ethylenediamine) in EtOH at room temperature for 15–45 min in high yields (85–98%) and purity *via* an easy work-up procedure (Scheme 25). A plausible mechanism for the Fe_3_O_4_@TDSN–Bi(iii) catalyzed synthesis of aminonaphthoquinones is depicted in Scheme 26. First, the aldehyde is activated by the catalyst to give 39 which upon nucleophilic attack of amine forms imine 40. Then, 2-hydroxynaphthalene-1,4-dione attacks imine 40 in the presence of the catalyst to furnish intermediate 41 desired products and releases the catalyst for the next run.^53^

### 2.4. Other-types of catalyzed reactions

**Scheme 27:**
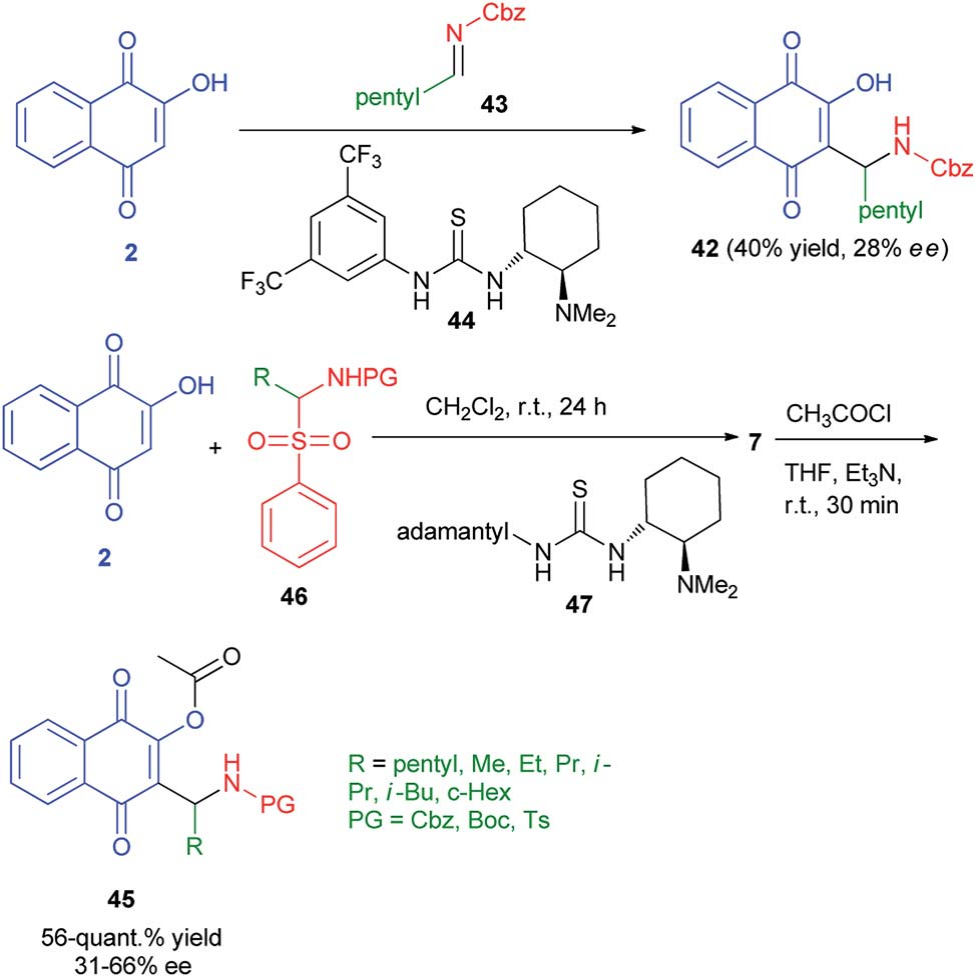
Preparation of Mannich bases 42 and 45.

Enantioenriched naphthoquinone Mannich base 42 by organocatalyzed nucleophilic additions of 2-hydroxy-1,4-naphthoquinone (2) to *in situ* formed imines has been reported by Westermann and Ayaz. In this process, lawsone (2) to preformed imine 43 using Takemoto's catalyst 44 (10 mol%) afforded the Mannich base 42 in only 40% yield and a very low enantioselectivity of 28% ee. Also, enantioenriched naphthoquinone Mannich bases 45 have been synthesized by the reaction of 2 with α-amidosulfone 46 in the presence of organocatalyst 47 in CH_2_Cl_2_ at room temperature for 24 h. Then, the reaction of title product with acetyl chloride in the presence of Et_3_N in THF at room temperature for 30 min gave the corresponding products 45 in 56-quant.% yield and 31–66% *ee* (Scheme 27).^54^

**Scheme 28:**
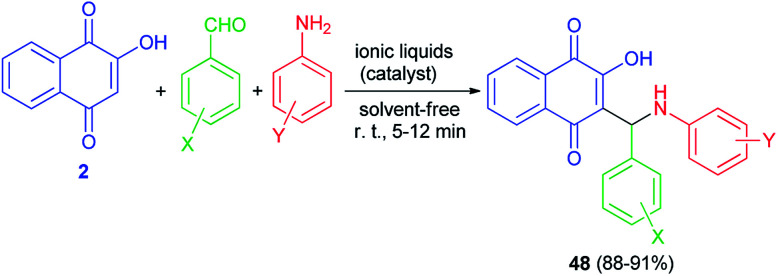
Ionic liquid catalyzed synthesis of aminonaphthoquinones 48.

Condensation of 2-hydroxynaphthalene-1,4-dione (2), anilines, and aromatic aldehydes in the presence of the mild basic ionic liquids, (A) DBU [CH_3_COO] (10 mol%), (B) Pyrr[CH_3_COO] (15 mol%), (C) Pyrr[HCOO] (15 mol%), (D) Pip[CH_3_COO] (10 mol%), (E) Pip[HCOO] (10 mol%), (F) [Hmim][HCOO] (15 mol%) and (G) 3-HPAA (15 mol%) as the most efficient catalysts with respect to the reaction time and yields under solvent-free and ambient conditions for 5–12 min afforded the corresponding aminonaphthoquinones 48 in high to excellent yields (Scheme 28).^55^ The proposed mechanism for the preparation of compounds 48 is similar to the first mechanism as indicated in Scheme 2.

**Scheme 29:**
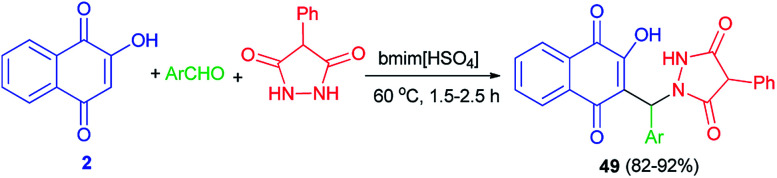
Task-specific ionic liquid catalyzed synthesis of naphthoquinone-urazole hybrids 49.

Khurana *et al.*^56^ described synthesis of naphthoquinone–urazole hybrids 49 in high yields (82–92%) *via* one-pot condensation of lawsone (2), various aromatic aldehydes having electron with-drawing and electron-releasing groups and 4-phenylurazole using task specific ionic liquid (bmim[HSO_4_]) (10 mol%) at 60 °C for 1.5–2.5 h (Scheme 29). A mechanistic rationale portraying the probable sequence of steps is similar to the second mechanism as shown in Scheme 2. All newly synthesized compounds were screened for *in vitro* antioxidant and anticancer activities against human breast (T47D), colon (HCT-15), lung (NCI-H522), liver (HepG-2) and ovary (PA-1) cancer cell lines. The *in vitro* antioxidant and anticancer activity of these compounds revealed that all of the synthesized naphthoquinone-urazole hybrids 49 have a significant activity. 3-Bromophenyl, 2-methylphenyl, 2-naphthyl and 3-methylphenyl derivatives were more active among all the naphthoquinone–urazole hybrids.

**Scheme 30:**
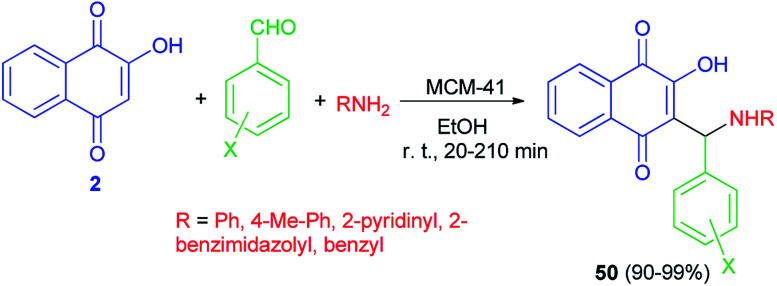
MCM-41 catalyzed synthesis of aminomethylnaphthoquinones 50.

Naimi-Jamal *et al.* succeeded in preparation of 2-hydroxy-1,4-naphthoquinone derivatives 50 from 2-hydroxynaphthalene-1,4-dione (2), aromatic aldehydes and heterocyclic or carbocyclic amines using MCM-41 nanoporous as catalyst in EtOH at room temperature for 20–210 min. The aromatic aldehydes carrying both electron-withdrawing and electron-releasing substituents were converted to their corresponding products in high yields (90–99%) (Scheme 30).^57^

**Scheme 31:**
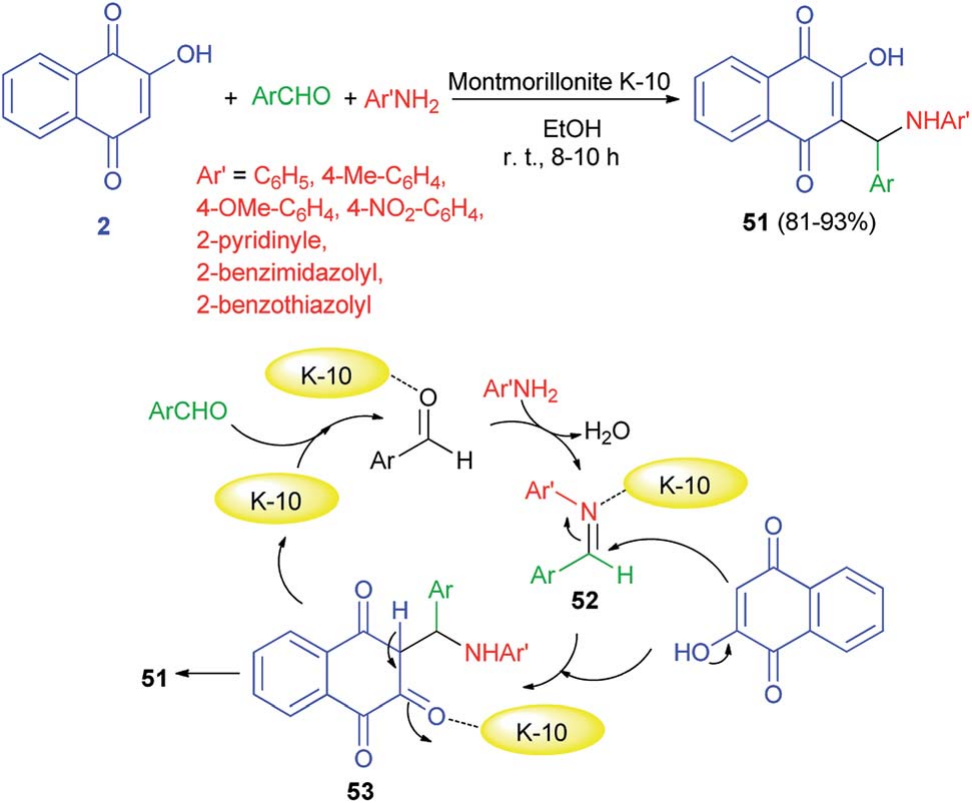
Montmorillonite K-10 catalyzed synthesis of aminonaphthoquinones 51.

An efficient one-pot protocol for the synthesis of 2-((substituted amino)(4-phenyl)methyl)-3-hydroxy-naphthalene-1,4-diones 51 has been developed by the three-component reaction of 2-hydroxynaphthalene-1,4-dione (2), aromatic aldehydes and anilines/heterocyclic amines using montmorillonite K-10 (10 mol%) as a catalyst in EtOH at room temperature for 8–10 h. Benzaldehydes and aromatic amines carrying both electron-donating and electron-withdrawing substituents and heterocyclic amines displayed high reactivity under this optimized conditions to afford the desired products in 81–93% yields. A speculative mechanistic explanation for this reaction is provided in Scheme 31. In the first step, aromatic amines attacked the activated benzaldehydes to form activated imines 52. The latter compounds reacted with lawsone, to give an intermediate 53 that underwent tautomerization to yield the products. Montmorillonite K-10 is likely to enhance the rate of Mannich reaction.^58^

**Scheme 32:**
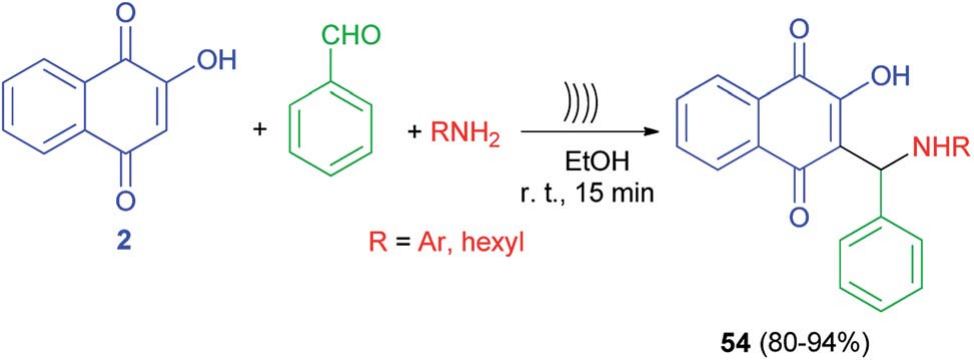
Synthesis of Mannich bases 54 derived from lawsone under ultrasonic irradiation.

Lopez-Lopez *et al.* developed a non-catalytic effective procedure to prepare Mannich base lawsone derivatives 54 in 80–94% yields using one-pot three-component reaction of lawsone (2), amines and benzaldehyde promoted by ultrasound irradiation at room temperature for 15 min (Scheme 32).^59^ A possible mechanism for the formation of 54 is proposed according to the first mechanism as indicated in Scheme 2.

**Fig. 3:**
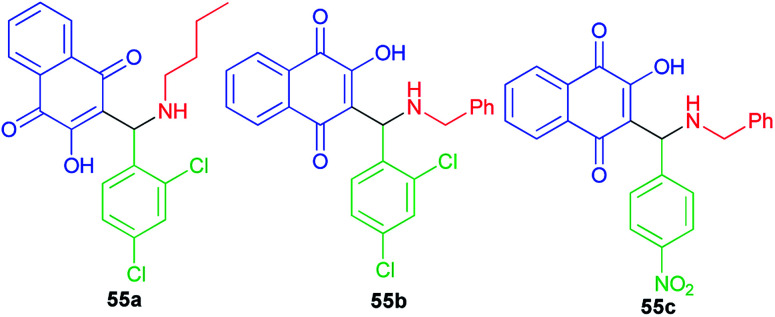
Structures of 3-aminomethylnaphthoquinones 55a–c active against HSV-1.

A small series of 3-aminomethylnaphthoquinones 55a–c active against HSV-1 with low in *vitro* and in *silico* toxicity profile has been evaluated by Paixao and co-workers. In spite of the highest antiviral activity against HSV-1 of compound 55b, the most promising molecule is probably compound 55a due to its grater SI value (SI = CC_50_/EC_50_). All compounds exhibited L-phase of lytic replication. Compounds 55a–b also inhibited IE and E phases to different degrees and they affect gD protein expression (L-phase). Structural features, such the nature of the substituent on the nitrogen atom (benzyl *versus n*-butyl) the conformation and LUMO distribution and energy profiles seem to modulate the antiviral activity of these compounds (Fig. 3).^28^

## 3. Synthesis of aminomethylnaphthoquinone Mannich base–metal complexes

### 3.1. Complexes derived from ferrocenylalkanamines

**Scheme 33:**
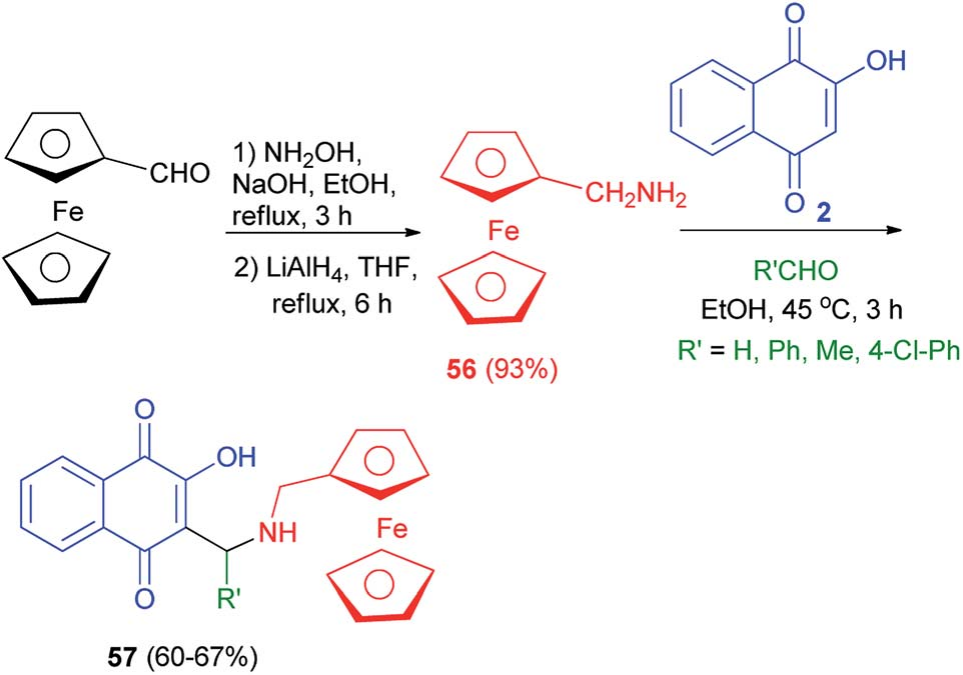
Synthsis of ferrocenyl aminohydroxynaphthoquinones 57.

Ferrocenylmethylamine 56 was first synthesized from ferrocene carboxaldehyde by condensation of hydroxylamine on the aldehyde function followed by a reduction with LiAlH_4_. The amine 56 was obtained in 93% global yield. Ferrocenyl aminohydroxynaphthoquinones 57, analogues of atovaquone, were synthesized from 2-hydroxyquinone, ferrocenyl amines 56 and aldehydes in EtOH at 45 °C for 3 h in 60–67% yields (Scheme 33).^60^

**Scheme 34:**
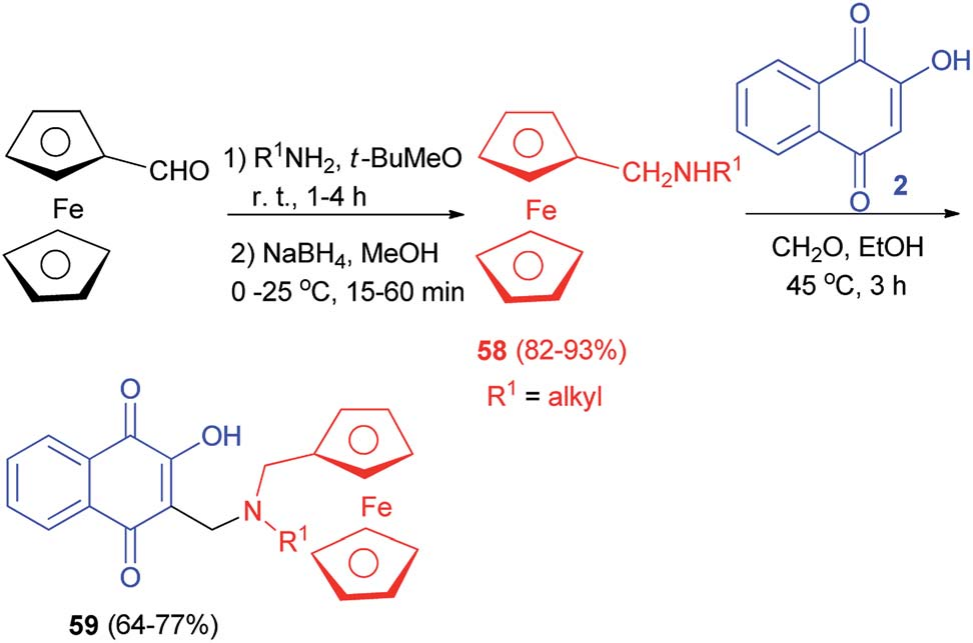
Synthesis of ferrocenyl aminohydroxynaphthoquinones 59.

Condensation of commercial amines on ferrocene carboxaldehyde followed by reduction with NaBH_4_ led to secondary ferrocenyl amines 58 in high global yield (82–93%). The ferrocenyl derivatives 59 were then prepared by condensation of lawsone (2) with the formaldehyde in the presence of the corresponding ferrocenylmethylamines 59 in EtOH at 45 °C for 3 h. Compounds 59 were obtained in 64–77% yields (Scheme 34).^60^ Ferrocenic atovaquone derivative 59 composed of the hydroxynaphthoquinone core plus an amino-ferrocenic group and an aliphatic chain with 6–8 carbon atoms were found to be significantly active against *T. gondii*. Moreover, this novel compound were also effective against the atovaquone-resistant strain of *T. gondii* (AtoR).^44^

**Scheme 35:**
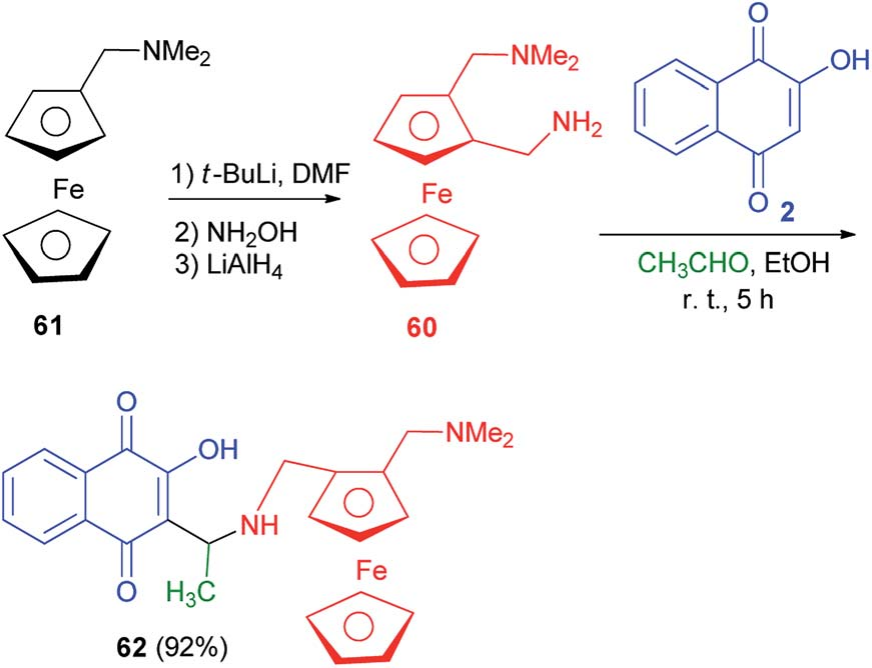
Synthesis of 3{*N*-(2-(*N*,*N*-dimethylaminomethyl)ferrocenylmethyl)-1-aminoethyl}-2-hydroxynaphthoquinone 62.

Following the procedure described in the literature,^60^ the diamine 60 was prepared from commercially available *N*,*N*-dimethylaminomethylferrocene (61). The treatment of compound 60 with 2-hydroxynaphthoquinone (2) in the presence of acetaldehyde in EtOH at room temperature for 5 h afforded the compound 62 as a mixture of diastereomers in 55/45 ratio (92% yield) which cannot be separated by silica gel column chromatography (Scheme 35).^60^

**Scheme 36:**
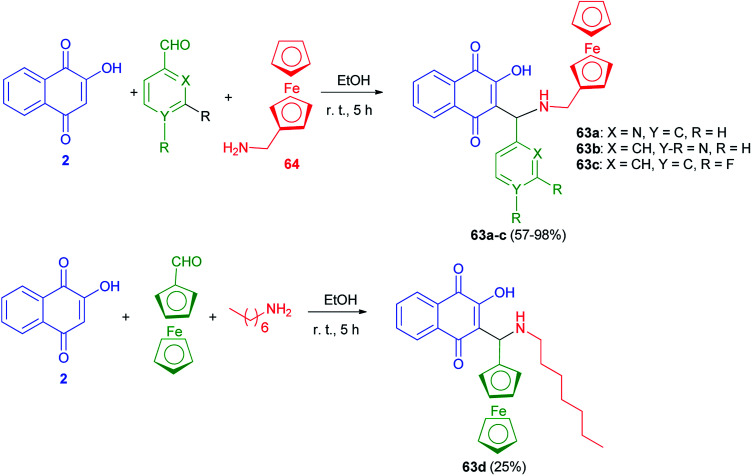
Ferrocene modified lawsone Mannich bases 63.

The new Mannich bases 63a–c were prepared in 57–98% yields *via* Mannich reaction of lawsone (2), aryl carboxaldehyde (2-pyridylcarboxaldehyde) for 63a, 4-pyridylcarboxaldehyde for 63b, and 3,4-difluorobenzaldehyde for 63c and ferrocene-1-ylmethylamine (64) in EtOH at room temperature for 5 h. Analogously, compound 63d was obtained in 25% yield from the reaction of 2 with ferrocene-1-yl carboxaldehyde and heptylamine in EtOH at room temperature for 5 h (Scheme 36). The 2-pyridyl derivative 63a was distinctly more active than its analogs 63b–d in breast, prostate and pancreatic cancer cells. Compound 63a also exhibited greater antiproliferative effects in the androgen-receptor negative PC-3 prostate and Pgp-expressing KB-V1/Vbl cervix carcinoma cell lines. Compound 63a reached sub-micromolar activities in these aggressive cancer cells and, thus, features a promising drug candidate for the efficient treatment of hormone- or multidrug-resistant cancer types.^22^

### 3.2. Copper complexes

**Scheme 37:**
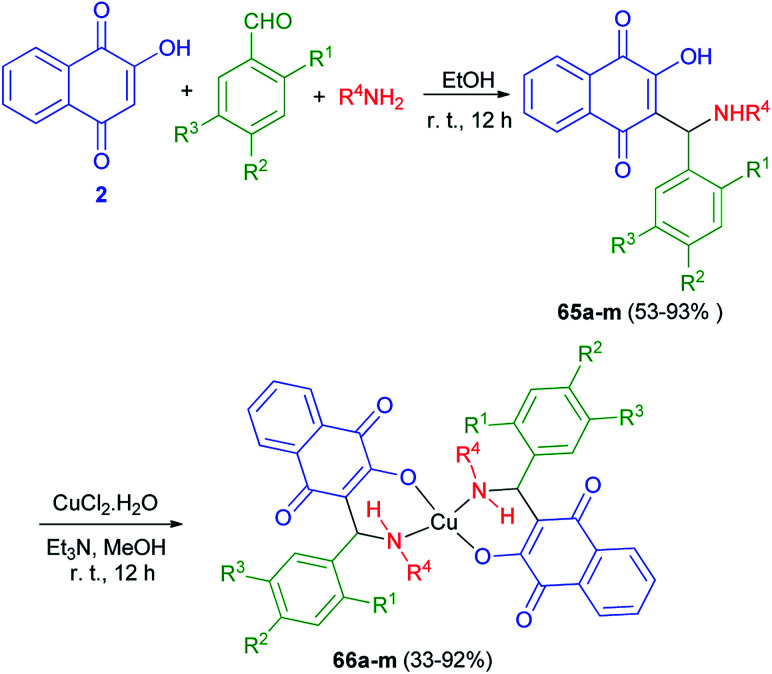
Synthesis of Mannich bases of 65 and their copper complexes 66.

A series of novel Mannich bases 65a–m derived have been synthesized in 53–93% yields from 2-hydroxy-1,4-naphthoquinone (2), substituted benzaldehydes and various primary amines (NH_2_R^4^, R^4^ = *n*-butyl, benzyl, allyl, 2-furfuryl) in EtOH at room temperature for 12 h in the dark. Followed, their Cu^2+^ complexes 66a–m have been prepared in 33–92% yields from the reaction of the compounds 65a–m with CuCl_2_.2H_2_O in MeOH in the presence of Et_3_N in the dark for 12 h at room temperature (Scheme 37). The antimicrobial activity of all compounds has been tested. In general, Mannich bases were more active than complexes, 65k (R^1^ = OH; R^2^ = H; R^3^ = Me; R^4^ = Bn) and 65m (R^1^ = OH; R^2^ = H; R^3^ = Br; R^4^ = Bn) being the most potent inhibitors. The MIC for the most active compound 65k against *S. Coli* was 20 μmol L^−1^ (8 μg mL^−1^), better than chloramphenicol (90 μmol L^−1^) and well below most values reported for other naphthoquinones.^25^

**Scheme 38:**
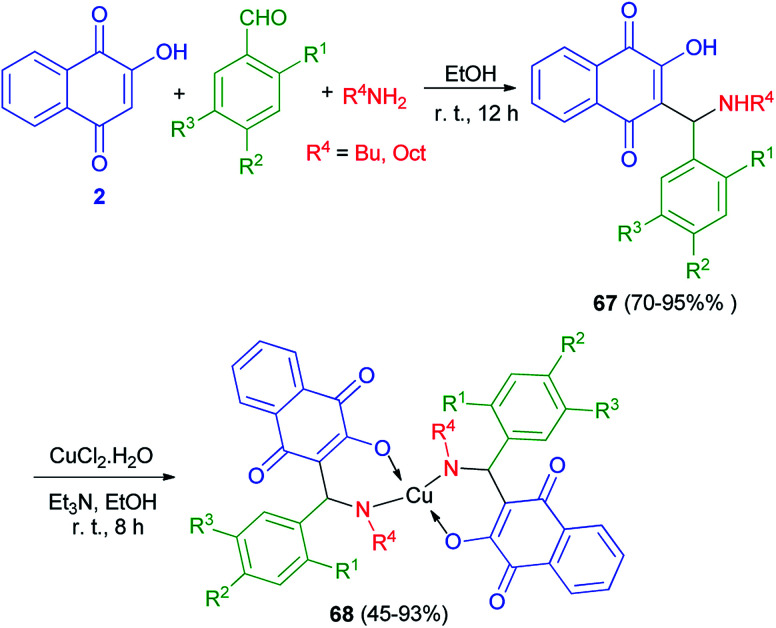
Synthesis of Mannich bases 67 and their copper complexes 68.

A new series of Mannich bases 67 derived from 2-hydroxy-1,4-naphthoquinone (2), substituted benzaldehydes and two primary amines, and their Cu^2+^ complexes 68 were synthesized by Cardoso and co-workers. At the first stage, 2-hydroxy-1,4-naphthoquinone (2) undergo an amino alkylation with butylamine or octylamine and benzaldehydes in EtOH at room temperature for 12 h, which led to lawsone derivatives 67 in high yields 70–95%. Complexes 68 were obtained by addition of triethylamine to an ethanolic suspension of compounds 68 and CuCl_2_·2H_2_O under stirring at room temperature for 8 h, with yields varying from 45 to 93% (Scheme 38). Compounds 68 evaluated for their potential as selective cholinesterase inhibitors (ChEIs). Eight copper complexes were identified and characterized as potent reversible and selective ChEIs with inhibitory potencies (IC_50_) and constants of inhibition (*K*_i_) ranging from 1.24 to 11.5 μmol L^−1^. One of the compounds was particularly promising, showing IC_50_ and *K*_i_ values of 1.24 ± 0.01 and 1.06 ± 0.01 μmol L^−1^, respectively, for huAChE.^26^

**Scheme 39:**
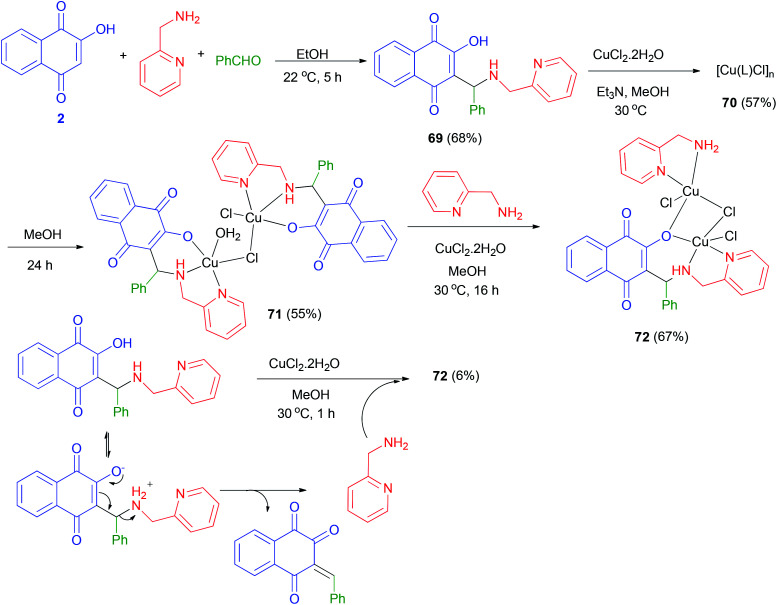
Synthesis of Mannich base 69 and their complexes 71 and 72.

A novel versatile tridentate 3-(aminomethyl)naphthoquinone proligand, 3-[*N*-(2-pyridylmethyl)aminobenzyl]-2-hydroxy-1,4-naphthoquinone (69), was obtained from the Mannich reaction of 2-hydroxy-1,4-naphthoquinone (2) with 2-aminomethylpyridine and yielded a polymeric compound 70 in 57% yield. When the reaction mixture was left stirring under the same conditions for over 5 h benzaldehyde in EtOH at 22 °C for 5 h in 68% yield. The reactions of 69 with CuCl_2_·2H_2_O in the presence of Et_3_N in MeOH at 30 °C yielded a polymeric compound 70 in 57% yield. When the reaction mixture was left stirring under the same conditions for over 5 h darkening was observed. Dark green solid 71 isolated after 24 h in 55% yield. The reaction of complex 71 with 2-aminomethylpyridine in using CuCl_2_·2H_2_O in MeOH at 30 °C for 16 h in afforded complex 72 in 67% yield. Complex 72 could also be obtained in 6% yield from decomposition of 69 in methanol using CuCl_2_·2H_2_O at 30 °C for 1 h (Scheme 39).^61^

### 3.3. Platinum complexes

**Scheme 40:**
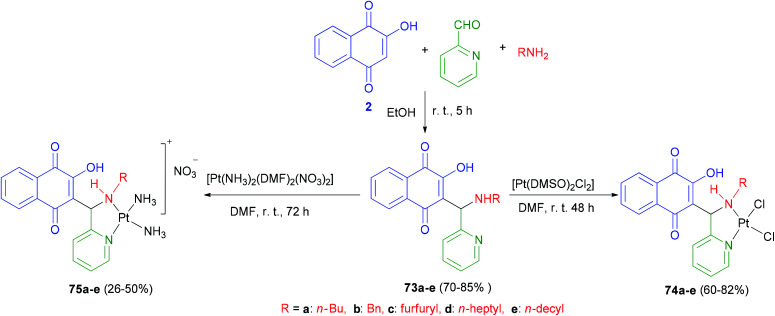
Synthesis of 3-(aminomethyl)naphthoquinones 73 and their Pt-complexes 74 and 75.

The first examples of platinum(ii) complexes of 3-(aminomethyl)naphthoquinone Mannich bases 73 have been synthesized by Vargas and coworkers. Proligands 3-[(R^1^-amino)(pyridin-2-yl)methyl]-2-hydroxy-1,4-naphthoquinones (R = *n*-Bu, 73a; Bn, 73b; furfuryl, 73c; *n*-heptyl, 73d and *n*-decyl, 73e) were synthesized in 70–85% yields from the Mannich reactions of lawsone (2) with the respective primary amines and 2-pyridinecarboxyaldehyde, in ethanol, under stirring, at room temperature for 5 h. The reactions of equimolar amounts of 73a–e and *cis*-[Pt(DMSO)_2_Cl_2_] in dimethylformamide (DMF) at room temperature for 48 h in the dark yielded the chlorido complexes *cis*-[ Pt(74a–e)Cl_2_] 74a–e in 60–82% yields as yellow solids (Scheme 40).

The amino complexes *cis*-[Pt(75a–e)(NH_3_)_2_]NO_3_75a–e were obtained as orange powders in 26–50% yields from the reactions of the solid proligands 73a–e with a solution of *cis*-[Pt(NH_3_)_2_(DMF)_2_](NO_3_)_2_ in DMF at room temperature in the dark for 72 h (Scheme 40). The cytotoxic activities of all compounds have been tested for different cancer cell lines. Proligands 73d and 73e have exhibited high activity against the tested cell lines, although they were only moderately active against the PC-3 cell line (IC_50_ = 29.9 and 15.6 mmol L^−1^, respectively). In general the compounds with the longest carbon chains (R = *n*-heptyl and *n*-decyl) have exhibited the highest activities.^62^

**Fig. 4:**
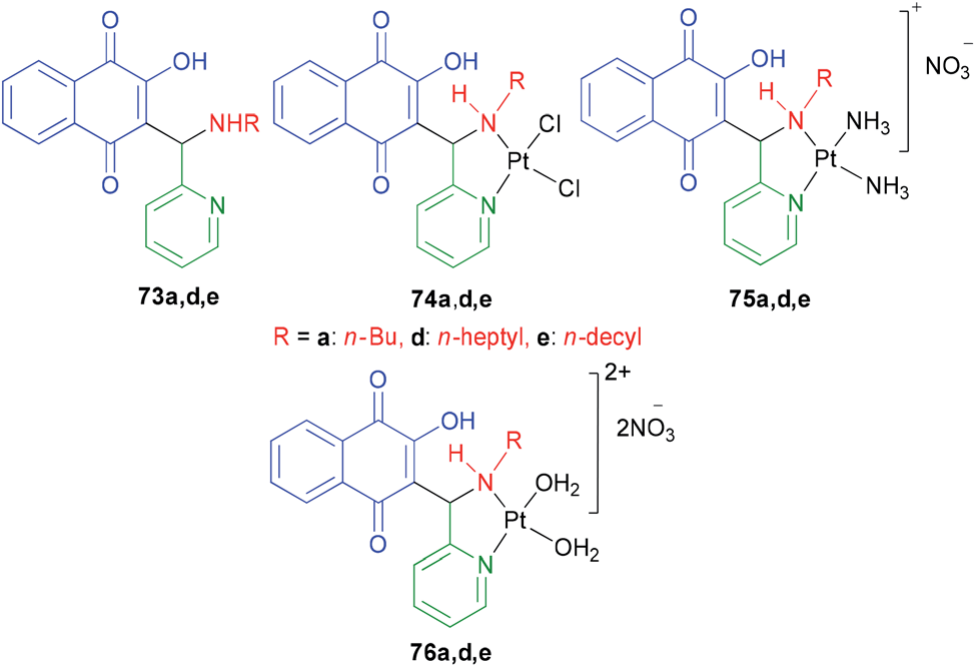
Structures of 2-hydroxy-3-(aminomethyl)-1,4-naphthoquinones 73 and their Pt^2+^ complexes 74, 75, 76a,d,e.

2-Hydroxy-3-(aminomethyl)-1,4-naphthoquinones 73a,d,e derivatives, chlorido 74a,d,e and amino 75a,d,e Pt^2+^ complexes were synthesized as described in the literature.^44^ The DMF solutions of compounds [Pt(73a,d,e)(DMF)_2_](NO_3_)_2_ were obtained by reacting a solution of AgNO_3_ with 73a,d,e in DMF at 50 °C for 24 h. [Pt(76a,d,e)(H_2_O)_2_](NO_3_)_2_76a,d,e were obtained by dilution of the DMF solutions with water to a final concentration of 95 : 5 (H_2_O : DMF). The water solution of [Pt(68a)(H_2_O)_2_](NO_3_)_2_76a was also prepared using the same procedure above, except for the use of H_2_O instead of DMF, for the gel mobility shift assays (Fig. 4). Several chlorido and amino Pt^2+^ complexes of 2-hydroxy-3-(aminomethyl)-1,4-naphthoquinone Mannich bases 73a,d,e exhibiting moderate to high cytotoxicity against cancer cell lines were studied in order to investigate their modes of DNA binding, *in vitro* DNA strand breaks, mechanism of topoisomerase (Topo I) inhibition and cellular accumulation. DNA model base studies have shown that complex 74a was capable of binding covalently to 9-ethylguanine (9-EtG) and 5′-GMP. The chlorido Pt^2+^ complexes 74a,d,e highly accumulate in prostate (PC-3) and melanoma (MDA-MB-435) cell lines, being able to induce DNA strand breaks *in vitro* and inhibit Topo I by a catalytic mode. On the other hand, the free 2-hydroxy-3-(aminomethyl)-1,4-naphthoquinones 73a,d,e and the amino Pt^2+^ complexes 75a,d,e neither cause DNA strand breakage nor exhibit strong DNA interaction, nevertheless the latter were also found to be catalytic inhibitors of Topo I at 100 μM. Thus, coordination of the Mannich bases 73a,d,e to the “PtCl_2_” fragment substantially affects the chemical and biophysical properties of the pro-ligands, leading to an improvement of their DNA binding properties and generating compounds that cleave DNA and catalytically inhibit Topo I. Finally, the high cytotoxicity exhibited by the free (uncomplexed) 2-hydroxy-3-(aminomethyl)-1,4-naphthoquinones 73a,d,e might be associated with their decomposition in solution, which is not observed for the Pt^2+^ complexes.^63^

**Scheme 41:**
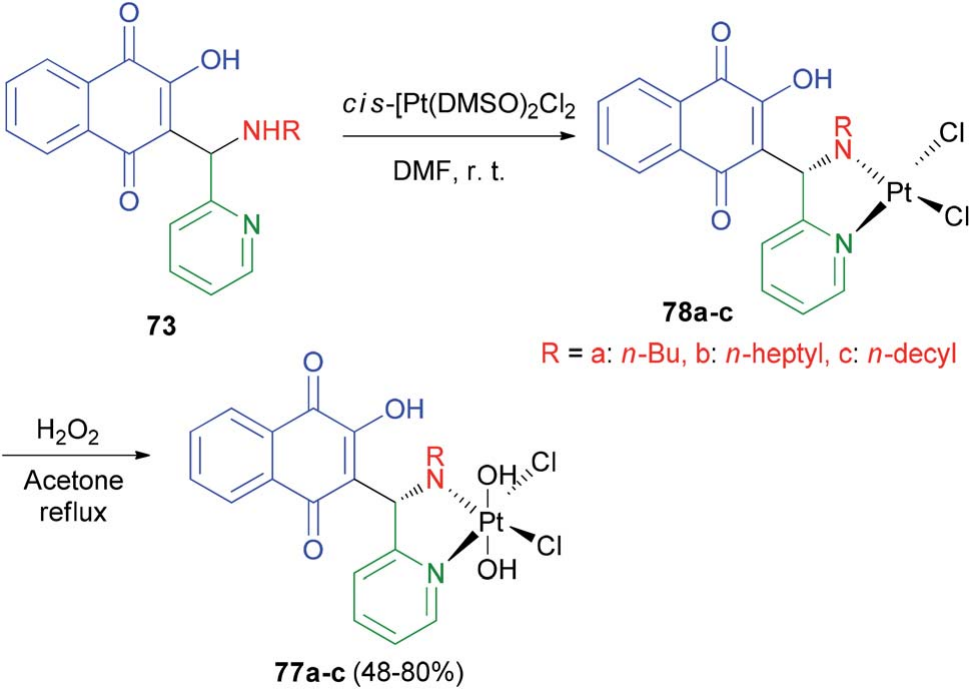
Synthesis of Mannich base-platinum(iv) complexes 77a–c.

Vargas *et al.* described synthesis of three novel platinum(iv) complexes *cis*,*cis*,*trans*-[Pt(77a–c)Cl_2_(OH)_2_] 77a–c (77 = 2-hydroxy-3-[(R-amino)(pyridin-2-yl)methyl]-1,4-naphthoquinone, R = *n*-butyl, 77a; *n*-heptyl, 77b and *n*-decyl, 77c). In this process, the yellow-orange complexes 77a–c were obtained by selective oxidation of 78a–c with H_2_O_2_ (30%) in refluxing acetone for 7 h in 48–80% yields (Scheme 41). The cytotoxicity studies against four human cancer cell lines have shown that in general the platinum(iv) and platinum(ii) derivatives exhibit the same cytotoxic profile and are all more active than cisplatin. The lowest *in vitro* IC_50_ values have been observed for 77b–c, which bear ligands with the largest R groups being the most lipophilic. Furthermore similar IC_50_ values for platinum(ii) and platinum(iv) complexes of the same ligands have been associated with rapid *in vitro* reduction of the latter complexes to afford 77a–c.^64^

### 3.4. Other-types metal complexes

**Scheme 42:**
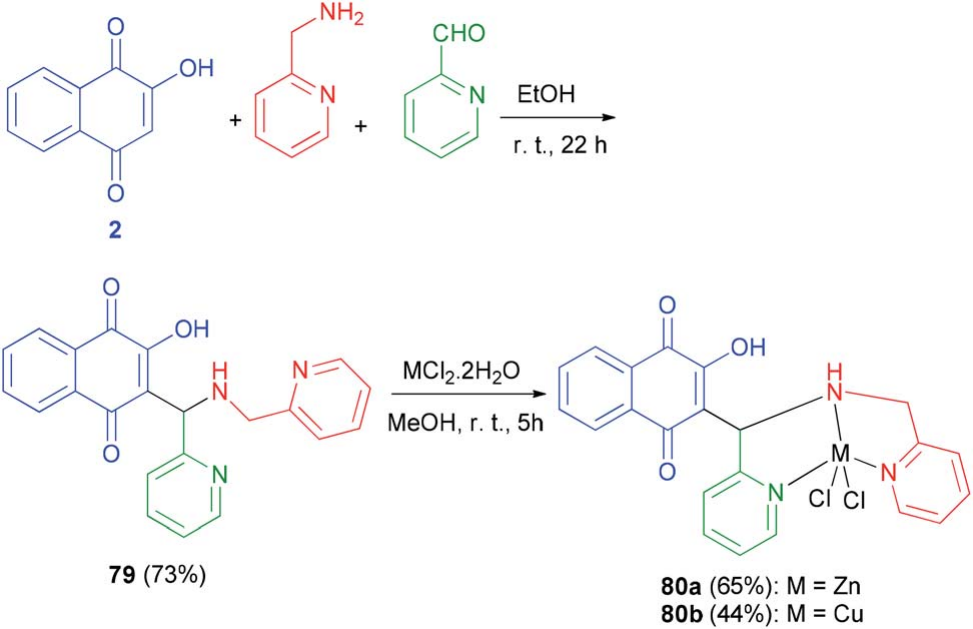
Synthesis of Mannich base 79 and complexes 80a–b.

Tridentate Mannich base 79 derived from lowsone, pyridine carboxyaldehyde and 2-aminomethylpyridine, have been synthesized in EtOH at room temperature in the dark for 22 h in 73% yield. Dichloro{3-[*N*-(2-pyridylmethyl)aminomethyl-2-pyridyl]-2-hydroxy-1,4-naphthoquinone}zinc(ii) 80a was obtained from the reaction of 79 with 1 equiv. ZnCl_2_·2H_2_O in methanol at room temperature in the dark for 5 h in 65% yield. The analogous dichloro 3-[*N*-(2-pyridylmethyl)aminomethyl-2-pyridyl]-2-hydroxy-1,4-naphthoquinone copper(ii) complex 80b was obtained from equimolar amounts of 79 and CuCl_2_·2H_2_O in methanol at room temperature for 5 h in 44% yield (Scheme 42).^65^

**Scheme 43:**
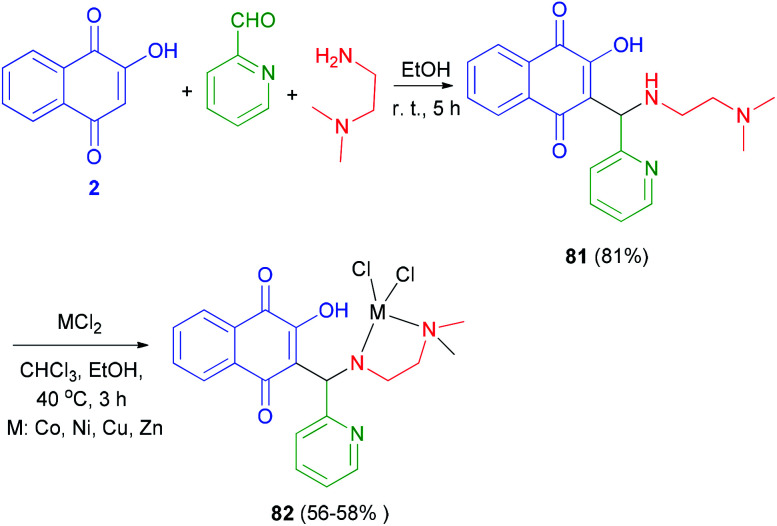
Metal(ii) complexes of Mannich base derived from lawsone 82.

The Mannich base ligand 81 has been synthesized in 81% from lawsone, pyridine-2-carbaldehyde and *N*,*N*-dimethylethylenediamine in EtOH at room temperature for 5 h. Ligand 81 was dissolved in chloroform and then corresponding metal chloride (Co(ii), Cu(ii), Ni(ii) and Zn(ii)) dissolve in ethanol was added drop wise and stirred at 40 °C for 3 h afforded metal(ii) complexes of Mannich base 82 in 56–58% yields (Scheme 43).^66^

## 4. Conclusions

Aminomethylnaphthoquinone Mannich bases derived from lawsone and their metal complexes belong to an important class of organic/organometallic compounds and exhibit a wide range of biological properties and due to their potent activities, thus thesynthesis of Mannich bases derived from lawsone is an area of current interest. Several multi-component reactions for the synthesis of aminomethylnaphthoquinones have been reported from lawsone, aldehydes and amines using green solvents, Bronsted and Lewis acid catalysts, ionic liquids, ultrasound irradiation under room temperature, reflux and solvent-free conditions. Also, Metal complexes of 3-(aminomethyl)naphthoquinone Mannich bases have been synthesized from lowsone, aldehydes and organometallic amines or the reaction of Mannich bases with metal cations under different conditions. This review summarizes the most multi-component strategies in the synthesis of aminomethylnaphthoquinone Mannich bases derived from lawsone and their metal complexes and applications.

## Conflicts of interest

There are no conflicts to declare.

## Supplementary Material
